# Isomeric Aromatic Polyimides Containing Biphenyl Moieties for Gas Separation Applications

**DOI:** 10.3390/polym15061333

**Published:** 2023-03-07

**Authors:** Laura Matesanz-Niño, David Cuellas, Carla Aguilar-Lugo, Laura Palacio, Alfonso González-Ortega, José G. de la Campa, Cristina Álvarez, Ángel E. Lozano

**Affiliations:** 1 Department of Macromolecular Chemistry, Institute of Polymer Science and Technology, ICTP-CSIC, Juan de la Cierva 3, E-28006 Madrid, Spaincristina.alvarez@ictp.csic.es (C.Á.); 2 SMAP, UA-UVA_CSIC, Research Unit associated to CSIC, Faculty of Science, University of Valladolid, Paseo Belén 11, E-47011 Valladolid, Spain; 3 Department of Organic Chemistry, Faculty of Sciences, University of Valladolid, Paseo Belén 7, E-47011 Valladolid, Spain; 4 Faculty of Chemistry, National Autonomous University of Mexico, Cd. University, Coyoacán, México 04510, Mexico; 5 UI CINQUIMA, University of Valladolid, Paseo Belén 5, E-47011 Valladolid, Spain

**Keywords:** aromatic polyimides, isomeric polymers, biphenyl monomers, thermal resistance polymers, gas separation

## Abstract

An optimized synthesis of the monomer 2,2′3,3′-biphenyltetracarboxylic dianhydride, iBPDA, was performed to obtain high molecular weight polymers. This monomer has a contorted structure that produces a non-linear shape, hindering the packing of the polymer chain. Aromatic polyimides of high molecular weight were obtained by reaction with the commercial diamine 2,2-bis(4-aminophenyl) hexafluoropropane, 6FpDA, which is a very common monomer in gas separation applications. This diamine has hexafluoroisopropylidine groups which introduce rigidity in the chains, hindering efficient packing. The thermal treatment of the polymers processed as dense membranes had two targets: on the one hand, to achieve the complete elimination of the solvent used, which could remain occluded in the polymeric matrix, and on the other hand to ensure the complete cycloimidization of the polymer. A thermal treatment exceeding the glass transition temperature was performed to ensure the maximum degree of imidization at 350 °C. The good mechanical properties of these materials allow for their use in high-pressure gas purification applications. Moreover, models of the polymers exhibited an Arrhenius-like behavior characteristic of secondary relaxations, normally associated with local motions of the molecular chain. The gas productivity of these membranes was high.

## 1. Introduction

Aromatic polyimides have gained notable importance in advanced technologies because of their excellent balance of mechanical, solvent-resistant, and thermal properties. As a result, this family of polymers has achieved a crucial role in the electronic industry, aerospace industry, and separation applications [1,2,3]. For this latter application, the polymers used as materials must be designed with a controlled balance of properties such as processability, glass transition temperature, and high fractional free volume [1,4,5,6,7,8,9]. In addition, all these polymer materials have to be amorphous and show relatively high transition temperatures, behaving as glassy materials at working temperatures [1,10,11,12,13].

In particular, gas separation membranes are a valid alternative to other established industrial separation processes, mainly due to their simplicity, continuous working, low energy consumption, and capital costs [14,15,16,17,18].

Nowadays, there is an industrial necessity for new gas separation membranes with high permeabilities (gas flux) and excellent permselectivities (separation factor) to be applied in industrial separations [19,20,21]. A high selectivity leads to a higher purity of products and allows a reduction in the number of operation steps, whereas permeability increases the velocity of the separation process [22]. However, it is well known that there is a trade-off between permeability and selectivity that restricts the gas separation [23,24,25,26,27].

One approach to achieving new polymeric materials with enhanced gas permeability is to include bulky structures, mainly pendant groups, which increase the fractional free volume, FFV [28,29,30]. In addition, using structures with high rigidity that hinders chain mobility produces a strong size-sieving ability [31]. Combining these two proposals should bring about materials with both high permeability and selectivity [26,32]. 

McKeown developed a new strategy to circumvent the commented trade-off between permeability and selectivity. His proposal consists of using polymers that have very contorted structures, which impart high rigidity to the macromolecular chain [30]. As a result, these so-called Polymers with Intrinsic Microporosity, PIMs, show very high permeabilities similar to rubbery polymers or glassy polymers such as poly(1-trimethylsilyl-1-propyne) (PTMSP), along with competitive gas selectivities [33,34,35,36,37,38]. 

This work has evaluated the gas separation properties of polyimides derived from the monomer 2,2′3,3′-biphenyltetracarboxylic dianhydride, iBPDA. The iBPDA biphenyl monomer has a contorted structure owing to the steric interaction between the two carbonyls of the anhydride moiety (imide on the polymer) and the 2′ and 6′ groups of the other phenyl group. This interaction produces a stiff non-linear shape that efficiently hinders the polymer chain’s packing, producing an increase in permeability along with good selectivity.

**Figure 1:**
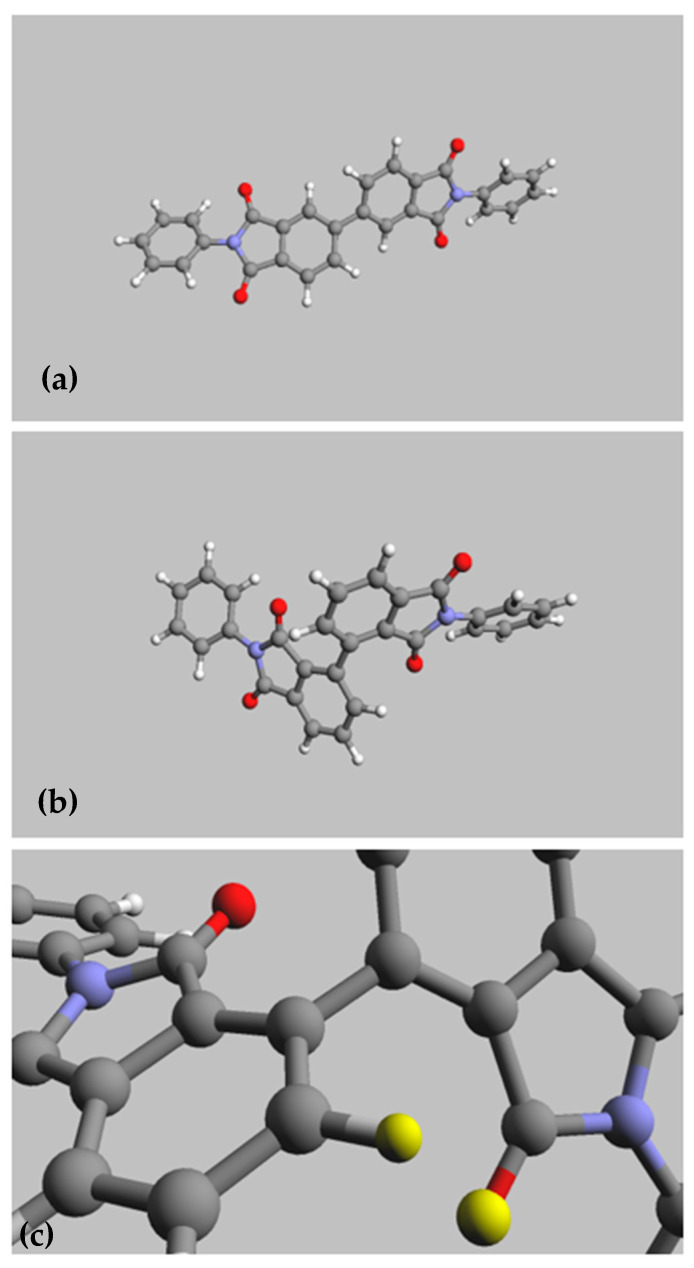
Molecular structure (AM1 semiempirical method) of the models made by reaction of aniline, An, with a biphenyl dianhydride: (**a**) BPDA-An, (**b**) iBPDA-An, and (**c**) scheme of the iBPDA-An model showing the steric interaction (yellow atoms) between an oxygen of the imide group and a hydrogen placed on 2-position of the biphenyl moiety.

To confirm the interactions mentioned above, molecular structures of biphenyl models were calculated with quantum-semiempirical methods and are displayed in Figure 1. 

After synthesizing iBPDA, it was polymerized with the 2,2-Bis(4-aminophenyl)hexafluoro-propane diamine, 6FpDA, which is very often used in the gas separation field because it produces polymer materials with enhanced gas separation properties [39,40,41,42,43,44]. Additionally, a set of isomeric copolyimides obtained by the combination of iBPDA and the 3,3′4,4′- biphenyltetracarboxylic dianhydride, BPDA [39], were obtained and evaluated, with the idea of modulating the gas separation properties induced by the iBPDA monomer [45,46].

## 2. Materials and Methods

The 3,3′,4,4′-biphenyltetracarboxylic dianhydride (BPDA) was purchased from Chriskev Company Inc., Overland Park, KS, USA, and purified by sublimation under high vacuum (220 °C at 0.05 mmHg). Diamine 2,2-bis(4-aminophenyl) hexafluoropropane (6FpDA) was also obtained from Chriskev Company Inc and purified by sublimation under high vacuum (170 °C at 0.05 mmHg). 

N,N-dimethylacetamide (DMAc), m-cresol, N-methyl-2-pyrrolidinone (NMP), tetrahydrofuran (THF), chloroform (CHCl_3_), N,N-dimethylformamide (DMF), and other chemicals and solvents were purchased at their highest purity grade and used without any further purification unless commented in the text.

### 2.1. Synthesis of Monomers

#### 2.1.1. Synthesis of 2,2′3,3′-Biphenyltetracarboxylic Dianhydride (iBPDA)

**Scheme 1:**
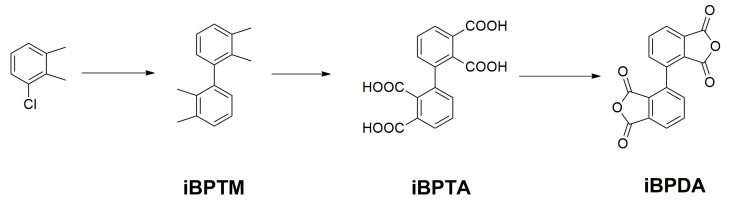
Synthesis of 2,2′3,3′-biphenyltetracarboxylic dianhydride, iBPDA.

The synthesis of 2,2′3,3′-biphenyltetracarboxylic dianhydride was carried out in a 3-step process starting from 2,3-dimethylchlorobenzene, as depicted in Scheme 1. The monomer iBPDA was obtained in the conditions described by Colon [47,48,49]. It began with a homocoupling reaction of 2,3-dimethylchlorobenzene, using the Dichlorobis(triphenylphosphine)nickel(II) as catalyst and zinc as an activator to reduce Ni(II) to Ni(0), which is the active species that produces the sp^2^-sp^2^ coupling. Subsequently, an oxidation process was carried out, using potassium permanganate in a basic medium to obtain the tetraacid compound with excellent purity. Finally, to ensure a high-purity iBPDA monomer, the tetraacid precursor was subjected to sublimation. This monomer was able to produce high molecular weight polyimides.

#### 2.1.2. Preparation of 2,2′,3,3′-Tetramethylbiphenyl (iBPTM)

A three-neck flask with a condenser and magnetic stirring was charged with dry sodium iodide (178.5 mmol, 26.90 g), triphenylphosphine (128.5 mmol, 33.68 g), Zn (546.3 mmol, 35.70 g), and Dichlorobis(triphenylphosphine)nickel(II) (17.8 mmoles, 11.73 g) in anhydrous DMF (270 mL) under N_2_ atmosphere. The reaction mixture was heated at 90 °C to activate the catalyst. Finally, 3-chloro-o-xylene (357.0 mmol, 50.00 g), dissolved in DMF (90 mL), was added to the mixture, and the reaction was maintained at 90 °C for 24 h. 

Afterward, the mixture was cooled to room temperature, and the formation of a precipitate was observed. Analysis of the solid indicated that it was a mixture of inorganics and the target compound that was recovered by filtering under a vacuum, dissolving with hot hexane, and finally concentrating to dryness. On the other hand, the DMF solution was treated with H_2_O_2_ solution (20 vol) to oxidize the triphenylphosphine and rotoevaporated to dryness, and the resulting solid was subjected to Soxhlet extraction with hexane. The hexane solution was concentrated to dryness, yielding the product 2,2′,3,3′-tetramethylbiphenyl with a high degree of purity. The overall yield was 90% (pf: 96 ± 1 °C). NMR spectra are shown in the supporting information section (Figures S1–S3). ^1^H-NMR, 300 MHz, CDCl_3_: 7.16 (m, 4H, H_c_, H_d_), 6.99 (dd, 2H, H_b_, JH_b_-H_c_ = 2.1 Hz, JH_b_-H_d_ = 6.8 Hz), 2.36 (s, 6H, H_f_, CH_3_), 1.98 (s, 6H, H_h_, CH_3_). ^13^C-NMR, 75 MHz, CDCl_3_: 142.3 (C_e_), 137.1 (C_g_), 134.5 (C_a_), 128.5 (C_c_), 127.2 (C_b_), 125.0 (C_d_), 20.5 (C_f_), 16.4 (C_h_). Elemental microanalysis: Calculated: C: 91.37, H: 8.36%. //Experimental: C: 91.08%, H: 8.51%.

#### 2.1.3. Preparation of 2,2′,3,3′-Biphenyltetracarboxylic Acid (iBPTA)

In a three-neck flask with a condenser and mechanical stirring, 2,2′,3,3′-tetramethylbiphenyl (54.7 mmol, 11.5 g), H_2_O/Pyr (2/1) (367 mL) and KMnO_4_ were added and the solution was heated to 100 °C. Subsequently, nine equal portions of KMnO_4_ (146 mmoles per portion, 23.0 g) were added at time intervals (30–45 min) to avoid intense reflux. Afterwards, the reaction was hot-filtered and the liquid was evaporated until around 70% of the solvent was eliminated. The resulting solution was acidified with 37% HCl until acid pH (pH = 2 approx.), and the formation of a precipitate observed. The precipitate was collected and purified by recrystallization in distilled water. The tetraacid compound iBPTA was obtained in 56% yield. pf: 273–275 °C (DSC). The NMR characterization is shown in the supporting information section (Figures S4–S6). ^1^H-NMR, 300 MHz, DMSO-d_6_: 7.85 (dd, 2H, H_d_, JH_d_-H_c_ = 7.8 Hz, JH_d_-H_b_ = 1.0 Hz), 7.51 (t, 2H, H_c_, JH_c_-H_b_ = JH_c_-H_d_ = 7.8 Hz), 7.40 (d, 2H, H_b_, JH_c_-H_b_ = 7.8 Hz). ^13^C-NMR, 75 MHz, DMSO-d_6_: 168.3 (C_f_), 167.0 (C_h_), 137.5 (C_g_ y C_e_), 133.4 (C_b_), 128.5 (C_c_), 129.0 (C_a_), 128.3 (C_d_), 127.5 (C_c_). Elemental Microanalysis. Calculated: C: 59.19, H: 3.05%. //Experimental: C: 58.97%, H: 3.16%.

#### 2.1.4. Preparation of 2,2′,3,3′-Biphenyltetracarboxylic Dianhydride (iBPDA)

The iBPTA tetraacid compound was sublimated at 240 °C under a high vacuum (0.05 mmHg). The crystalline solid deposited on the cold finger was collected to afford the corresponding dianhydride with a quantitative yield (pf: 269 °C). The NMR characterization is shown in the supporting information section (Figures S7–S9). ^1^H-NMR, 500 MHz, DMSO-d_6_: 8.21(dd, 2H, H_d_, JH_d_-H_c_ = 7.1 Hz, JH_d_-H_b_ = 1.8 Hz), 8.12 (t, 2H, H_c_, JH_c_-H_b_ = 7.8 Hz), 8.09 (d, 2H, H_b_). ^13^C NMR, 125 MHz, DMSO-d_6_: 162.6 (C_f_), 162.3 (C_h_), 138.0 (C_b_), 136.1 (C_c_), 134.2 (C_e_), 121.6 (C_g_), 128.5 (C_a_), 125.9 (C_d_). Elemental Microanalysis: Calculated: C: 65.32%, H: 2.06%. //Experimental: C: 64.97, H: 1.79%. 

### 2.2. Polyimide Synthesis

General polymerization was carried out as follows [1,6]:

In a three-neck flask equipped with a mechanical stirrer and an inert atmosphere, 6FpDA (10 mmol) was added. Subsequently, m-cresol (1 mL per mmol of diamine) and the corresponding dianhydride (or the dianhydride mixture) (10 mmol) were added [48]. The solution was then heated to 50 °C for 2 h. After that, anhydrous pyridine (2 mmol per mmol of diamine) was added, then the reaction was heated at 80 °C for 2 h, at 100 °C for another 2 h and finally at 120 °C. At this point, benzoic acid was added (2 mmol per mmol of diamine) and the reaction was left at this temperature for 10 h. After this time, the imidization step was accomplished by heating the solution at 180 °C for 9 h. Finally, the resulting polymers were precipitated in ethanol/water (1/1), extracted with the same solvent in a Soxhlet for 24 h, and then dried in an oven at 80 °C under vacuum. Yields of over 97% were obtained in all cases.

### 2.3. Preparation of Polymer Films

Polymer films were prepared by a solution-evaporation (casting) process from 10% (*w*/*v*) polymer solutions in CHCl_3_. The solutions were filtered through a 3.1 μ Symta^®^ fiber-glass syringe filter and deposited onto a level glass. Subsequently, the solvent was allowed to evaporate at room temperature for 24 h.

Once the film was formed, it was subjected to a thermal protocol to eliminate as much solvent as possible, heating at 120 °C for 3 h and at 160 °C for a further 6 h in a vacuum oven. As a result, polyimide films with homogeneous thicknesses between 30 and 50 μm were obtained for each aromatic polyimide.

### 2.4. Characterization Methods

Differential Scanning Calorimetry (DSC): The measurements were performed in a TA-DSC Q2000 differential scanning calorimeter, analyzing approximately 5–10 mg of the polymer film in a nitrogen atmosphere (flow rate of 50 mL min^−1^) with a heating rate of 20 °C min^−1^. All samples were double-scanned up to 400 °C. The first cycle was used to homogenize the thermal history of the samples and ensure the elimination of residual stresses that may have occurred during the precipitation and polymer purification phases. The Tg was determined in the second heating cycle, taking as a value the midpoint temperature of the (1/2ΔCp) [49] of the interval that is limited by the tangents drawn before and after the change in the sample’s heating capacity with temperature.

Thermogravimetric analysis (TGA): These analyses were performed in a Thermal Analysis (TA) TG Q500 thermobalance, in samples of approximately 5–10 mg, under a flow of 40 mL min^−1^ of Nitrogen, from 40 to 800 °C. A high-resolution method (HiRes) was used, implying high heating speeds when the system does not detect weight changes, while the speed becomes very low or even zero when losses are detected. This method detects overlapping processes and separates them into their individual components. In some instances, and to eliminate some results derived from the HiRes method, dynamic thermogravimetric analyses were performed at 10 °C min^−1^ under N_2_. The onset degradation temperature (Td) was taken as the temperature at which the polymer began to degradate and the char yield was the TGA residue (%) at 800 °C under a N_2_ atmosphere. The glass transition temperature (Tg) was determined as the temperature in the middle point of the endothermic step during the second scan of DSC measurements.

Mechanical properties: Traction tests were carried out using the constant speed deformation technique, so the polymer responds with a resistance, which is recorded in units of force, to the deformation to which it is subjected. From the relationship between stress and deformation, the value of Young’s modulus (E, GPa), the tensile strength (MPa) at the point of break and the strain at break (%) were obtained. The tests were carried out on 5 mm wide and 3 cm long films in a vertical extension dynamometer MTS Synergie 200 Universal Testing Machine, using mechanical clamps with a distance between them of 10 mm and applying an extension speed of 5 mm min^−1^. 

X-ray Diffraction (WAXD): X-ray measurements were performed on the membranes placed on an aluminum disc, with a Bruker D8 X-ray diffractometer Advance, operating at the speed of 1° min^−1^ from 2θ equal to 3° to 35° and using Nickel filtered K-Cu radiation (λ = 1.5460 Ǻ).

Solubility: Solubility tests were carried out in a test tube, where 1–2 mg of polymer and 1 mL of solvent were added. The system was subjected to magnetic agitation for 24 h. In case of nothing dissolving, it was heated at the boiling point of the solvent. The solvents used were DMAc, m-cresol, NMP, THF, and CHCl_3_.

The determination of η_inh_ was carried out experimentally by measuring the fall time along the capillary, using polymer solutions at 0.5% concentration in NMP, in an Ubbelohde viscometer in a thermostated bath at 25 °C. 

Molecular weights of polymers (M_w_ and M_n_) were determined by size exclusion chromatography (SEC). The experiments were carried out on a Perkin Elmer system consisting of a Perkin Elmer LC PUMP 250 pump providing a working flow rate of 1 mL min^−1^, a Perkin Elmer LC OVEN 101 oven, and a Philips Pye Unicam PU 4025 UV detector. Two Polymer Labs Resipore columns were used (3 m, 300 mm × 4.6 mm, molecular weight detection range from 200 to 400.000 g mol^−1^). The experimental working conditions were the following: wavelength of 275 nm, oven temperature of 70 °C, and solvent flow of 0.3 mL min^−1^. Calibration was carried out with low polydispersity polyethylene glycol (Fluka) standards for an Mw range from 200 to 500.000 g mol^−1^. The sample was prepared by dissolving 1 mg of polymer in 1 mL of DMF with LiBr. This salt was incorporated to eliminate associations of polymer chains with the solvent.

Density and fractional free volume (FFV): The density (*ρ*) of membranes was determined using a top-loading electronic XS105 Dual Range Mettler Toledo balance coupled with a density kit based on Archimedes’ principle. The samples were sequentially weighed in air and into high-purity isooctane at room temperature. The density was calculated from Equation (1).

(1) $$\rho =\rho_{liquid}\;\times \;\frac{w_{air}-w_{liquid}}{w_{air}}$$

where *ρ_liquid_* is the density of isooctane, *w_air_* is the weight of the sample in air and *w_liquid_* is its weight when submerged in isooctane. Four density measurements were made for each sample. 

The density data were used to evaluate chain packing with the fractional free volume (FFV) calculated from the following Equation (2):

(2) $$FFV=\frac{V_{e}-1.3V_{w}}{V_{e}}$$

where *V_e_* is the specific volume of the polymer and *V_w_* is the polymer van der Waals volume calculated in the Biovia Materials Studio program [50]. A 20-unit polymer structure was built using the builder polymers, atom volumes and surface algorithms of the program Materials Studio, and was optimized by using the compass force field to determine the molecular volume.

Fourier Transform Attenuated Total Reflection Infrared Spectroscopy (ATR-FTIR): All spectra of membranes were recorded by a diamond-tipped attenuated total reflection (ATR) device. The measurements were made on a Perkin Elmer Spectrum One FT-IR Spectrometer, to which the Universal ATR Sampling Accessory module was attached. Four scans were used, in the interval of 4000 to 650 cm^−1^, with a resolution of 4 cm^−1^ to obtain the spectra with high quality. 

Elementary microanalysis: The elemental determination of the precursors, final products, and polymers was carried out in a Carlo Erba^®^ EA1108 total organic carbon elemental analyzer calibrated with acetanilide for the determination of C, H, and N.

The dynamomechanical properties, DMTA, were carried out in Polymer Laboratories with a DMTA MKII dynamomechanical thermal analyzer, measuring in tension, with a constant force of 0.3 N. The samples were prepared from the same membranes used for gas separation, with an approximate length of 22 mm, a width of 2 mm, and a thickness between 30 and 60 μ. Modulus and loss tangent were determined at 1, 3, 10, and 30 Hz frequencies, with a temperature sweep between −140 and 250 °C and a heating rate of 1.5 °C min^−1^.

### 2.5. Gas Separation Experiments

A barometric permeation method was used to determine steady state pure gas permeability at 30 °C, applying a pressure of 3 bars. This technique consisted of measuring the increase in gas pressure in a closed chamber caused by gas permeation through the membrane in a piece of equipment. The measurements were made using five gases in the following order: helium, oxygen, nitrogen, methane, and carbon dioxide.

From this equation, the membrane permeability was obtained, *P*:

(3) $$P=\frac{273.15}{76}\times \frac{Vl}{\pi r^{2}Tp_{o}}\times \frac{dp}{dt}10^{10}$$

where *V* is the volume of the low-pressure chamber, expressed in cm^3^, *l* is the thickness of the membrane in cm, *r*^2^ is the surface of the membrane in cm^2^, *T* the working temperature in K, *p_o_* is the pressure in the high zone in mbar, and dp/dt represents the slope of the line in mbar s^−1^. The factors included reference the results at standard pressure and temperature conditions (76 cm Hg and 273.15 K). The dimensional analysis of the equation indicates that P has Barrer as units (1 barrer = 10^−10^ (cm^3^(STP) cm cm^−2^ s^−1^ cm Hg^−1^)). 

The ideal selectivity, *α_A,B_*, between two gas species, A and B, is the ratio of the single gas permeabilities (Equation (4)).

(4) $$\alpha_{A,B}=\frac{P_{A}}{P_{B}}$$

The cut-off point of the extrapolation of the straight section of the graph with the abscissa gives us the time delay (*θ*), which is the time needed to reach the stationary state, after which the gas diffusion through the membrane is constant. From this time, D can be determined by means of the next equation.

(5) $$D=\frac{l^{2}}{6\theta }$$

The solubility coefficient, *S*, for the gas in the polymer was evaluated indirectly, assuming the validity of the diffusion–solution mechanism [51] (Equation (6)).

(6) $$S=\frac{P}{D}$$

## 3. Results and Discussion 

### 3.1. Polymer Synthesis and Characterization 

**Scheme 2:**
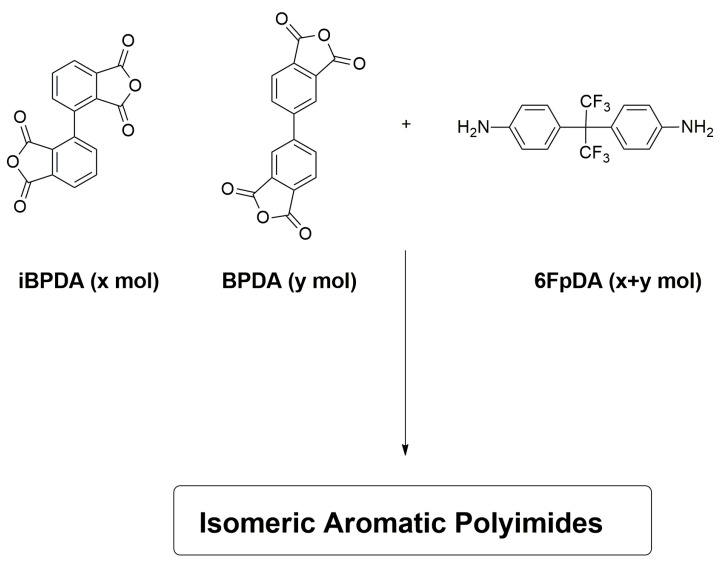
Synthesis of copolyimides from BPDA and iBPDA diahydrides and 6FpDA diamine.

Polyimides and copolyimides were synthesized by using a one-step polyimidization method (Scheme 2). This method involves using polar protic solvents such as m-cresol at high temperature. In addition, basic and acid catalysts were used to improve the monomer reactivity and, consequently, the molecular weight of the polymer. The use of two-step polyimidation methodologies, even using the in situ silylation method [52,53], produced polymers with lower molecular weight than those observed when single-step polyimidation synthesis was employed.

Polymers were characterized by NMR spectroscopy, FT-IR, DSC, and TGA. Molecular weights were determined by GPC and inherent viscosity measurements. The homo and copolymerization reactions, together with the acronyms that will be used in this work, are shown in Table 1, and the synthesis of the copolyimides is displayed in Scheme 2.

### 3.2. Thermal Treatment of Membranes

The thermal treatment of the polymers processed as dense membranes had two targets: on the one hand, to achieve the complete elimination of the solvent used, which could remain occluded in the membrane, and on the other hand, to ensure the complete cycloimidization of the polymer.

This type of treatment is usually carried out exceeding the glass transition temperature, since, at these temperatures, the conformational requirements to form the imide can be achieved. Thus, the following sequential heat treatment was performed to ensure the maximum degree of imidization and the removal of the possible solvent occluded in the prepared films: (a) 200 °C, 1 h in N_2_ atmosphere; (b) 250 °C, 30 min under vacuum; (c) 300 °C, 30 min under vacuum; (d) 325 °C, 15 min under vacuum; and (e) 350 °C, 12 min under vacuum.

### 3.3. Characterization of Polymers

The polymers were characterized by ^1^H-NMR, ^13^C-NMR, and heteronuclear correlation (HSQC-NMR), and the spectra, together with the signal assignment, are shown in the ESI of this report.

These aromatic polymers showed very good thermal properties, with Tg > 310 °C and initial degradation temperatures above 500 °C (see Table 2).

#### 3.3.1. ATR-FTIR Characterization

ATR-FTIR was performed for two different purposes: firstly for the characterization of the polymers, and then for seeing changes in the chemical structure due to the thermal treatment of membranes. The full ATR-FTIR spectra of polymers are shown in the supporting information section.

**Figure 2:**
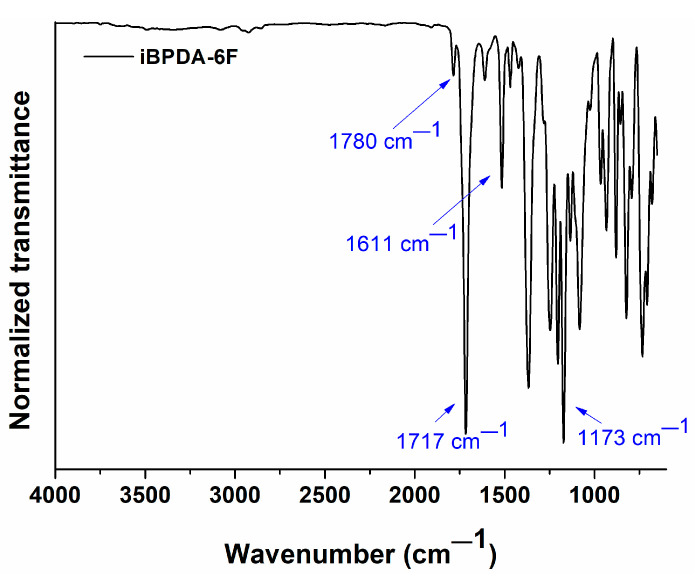
ATR-FTIR spectrum of iBPDA-6F homopolymer.

In the characterization of these materials, the absorption IR bands of the iBPDA-6F polymer at 1780, 1717, 1611, and 1175 cm^−1^ were assigned, respectively, to the asymmetric and symmetric C=O stretching vibrations, to the aromatic C-C stretching vibration, and to the C-F stretching vibration of the CF_3_ moieties (Figure 2) [54,55,56].

**Figure 3:**
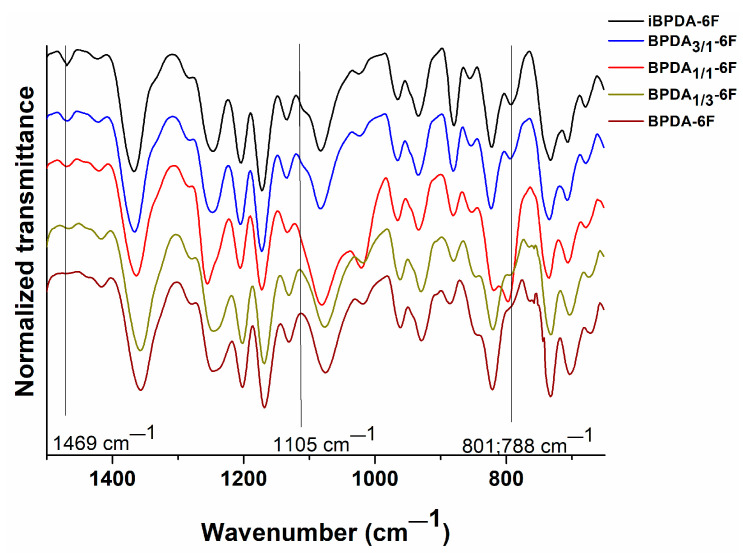
Influence of the percentage of both isomers, iBPDA and BPDA, on the ATR-FTIR absorption bands of polymers.

In Figure 3, the ATR-FTIR plot of all polymers is depicted for the better visualization of the bands between 1500 and 650 cm^−1^. It was observed that, as the proportion of BPDA monomer increased, the following events happened:

The disappearance of absorption band at 1469 cm^−1^ in the iBPDA-6F homopolymer, which moves to 1435 cm^−1^ in the BPDA-6F homopolymer. The peak at 1105 cm^−1^ diminished as the mole fraction of BPDA monomer increased. Both absorptions can be associated with aromatic C-H bond.

The disappearing of absorptions at 801 and 788 cm^−1^, which are associated with the *ortho* substitution of the aromatic system of the iBPDA dianhydride when the mole fractions of BPDA increased while presenting the appearance of a typical band of *meta* substitution at 793 cm^−1^.

**Figure 4:**
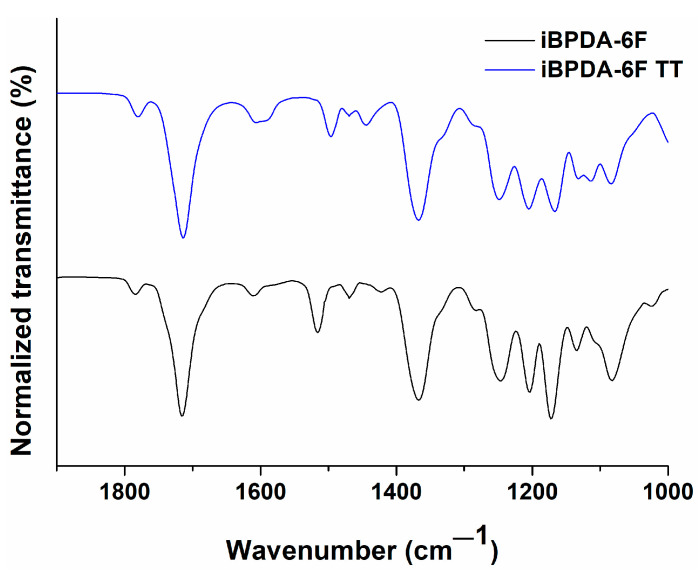
ATR-FTIR comparison of the untreated iBPDA-6F polymer and the film treated by the temperature protocol.

As mentioned, ATR-FTIR infrared spectroscopy was also employed to see the differences in the chemical structure of films before and after the thermal treatment. Figure 4 denotes the absence of the typical absorption band at 1680 cm^−1^ (C=O stretching vibration) assigned to polyamic acid units in the polymer structure, allowing us to conclude that the polymers were well imidized even before thermal treatment. The small differences in intensity observed in some bands cannot be associated with significant changes in the chemical structure.

#### 3.3.2. Inherent Viscosity and Molecular Weight of Polymers

The values of Table S2 (supporting information section) depict the inherent viscosity and the molecular weights determined by SEC. The results allowed us to ensure that the synthesis used to obtain the polymers was satisfactory, since the M_w_ and M_n_ values were high.

**Figure 5:**
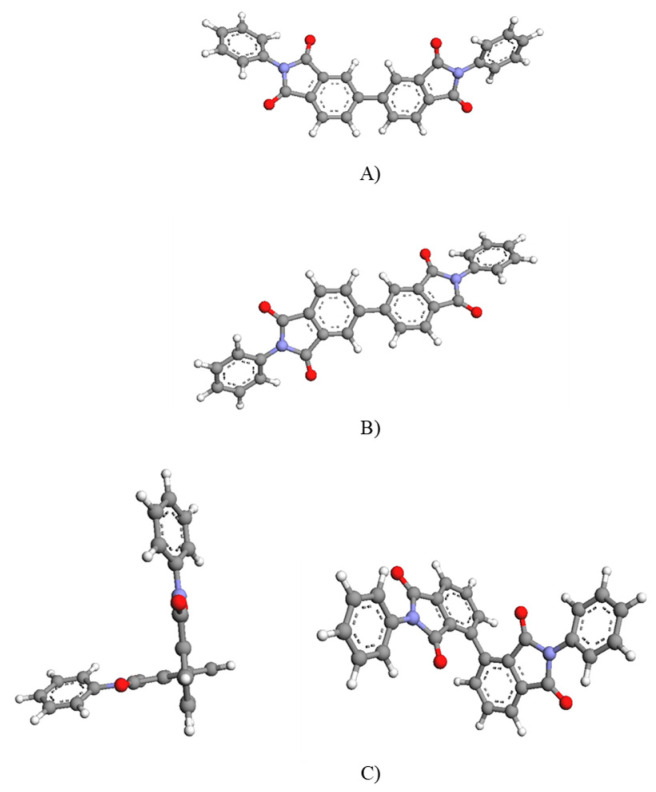
Main conformations of BPDA monomer (**A**,**B**) and iBPDA monomer (**C**). Minimization obtained by DFT B3LYP/6-31G(d).

It was observed that the polymers’ polydispersity values, PDI, were lower than those expected for polycondensation polymers, whose theoretical IP is 2. These lower values were plausibly due to the purification process, since the Soxhlet extraction carried out caused the removal of the more soluble low molecular weight species. Regarding the molecular weights, the values for all polymers were very similar. Therefore, strictly speaking it is logical to consider that the higher the viscosity, the higher the molecular weight; consequently, the BPDA-6F polymer would have a higher molecular weight. However, this result is only valid when the parameters of the Mark–Houwink equation are similar$\;(\eta =KM^{a}$). Thus, polymers with a rigid-linear structure (for example, aromatic polyamides derived from terephthalic acid, polybenzoxazoles, and polybenzothiazoles) have high values of parameter a, which could lead to higher viscosity values. Figure 5 shows the structural units of BPDA and iBPDA. It is observed that the structural unit of the iBPDA monomer is non-linear, whereas the structural unit of the BPDA monomer is more linear. Therefore, it is presumable that the BPDA-6F homopolymer should have higher viscosity at a similar degree of polymerization.

#### 3.3.3. Solubility

All of the polymers presented excellent solubilities (see Table S3) in polar aprotic solvents and *m*-cresol. However, they were also soluble in chloroform and tetrahydrofuran, in which many polyimides are generally not soluble. Among them, the polymer with the highest solubilities was the iBPDA-based homopolymer [45]. It should also be mentioned that the thermal protocol did not result in crosslinking of the material since the thermally treated polymers dissolved readily in chloroform.

In conclusion, the combination of a biphenyl structure and the hexafluoroisopropylidene group enables these polymers to be processed into films from low boiling point solvents [39].

#### 3.3.4. Density and Free Volume Fraction, FFV, of Polymers

The values of densities and FFV are displayed in Table S4 of the supporting information. Aromatic polyimides’ fractional free volume values, FFV, were greater than 0.180. These values are high since a polymer taken as a reference in gas separation studies, 6FDA-6FpDA (an aromatic polyimide with high permeability and excellent selectivity), shows an FFV value of 0.184. The maximum FFV value corresponded to the BPDA-6F polymer. Therefore, this polymer would be expected to give the highest gas permeability values. However, as discussed below, the highest permeability values correspond to the iBPDA-6F polymer. In addition, this polymer presented a much better balance between permeability and selectivity than that of BPDA-6F homopolymer or copolymers [25,57]. The explanation for this finding is not simple, and it could probably be due to differences in FFV distribution.

#### 3.3.5. X-ray Diffraction

**Figure 6:**
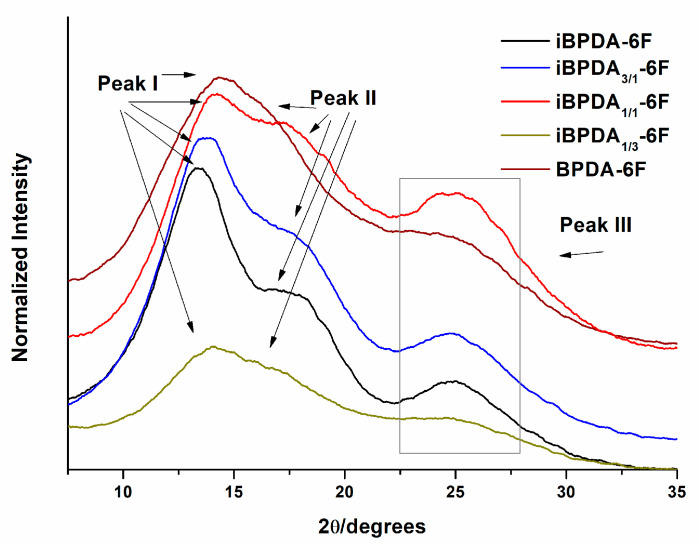
X-ray diffractograms (WAXS) of the synthesized polymers.

Figure 6 depicts the WAXS diffractograms of the polymers synthesized in this work. All polyimides showed an amorphous structure with two or three diffraction maximum peaks.

The d-spacings for the polymers were calculated by applying the Bragg equation (nλ=2dsenθ) to the peak values in the diffractograms.

The interest peaks for establishing correlations between structure and gas separation are peaks I and II. In contrast, peak III corresponds to a very low spacing, about 3.6 Ǻ, and is not useful for this purpose. The diffraction peak I, which appears in all polymers, gave us an idea of the packing ability of polymer chains. It could be seen that the d-spacing decreases as the proportion of BPDA monomer increases, going from 6.6 Ǻ in iBPDA-6F to 5.7 Ǻ in BPDA-6F. This behavior indicates that the introduction of BPDA resulted in a more compact polymer.

Peak II (d-spacing around 5.0 Ǻ) only appears in polyimides possessing the iBPDA monomer, which means that this monomer causes an ordering or packing of chains that are not present in the BPDA-derived polyimide. These findings indicate that at equal free volume fraction, the iBPDA homopolymer has larger free volume elements, and this could explain the higher permeability having a lower FFV. Many authors have correlated [58,59] the material’s permeability with the average d-spacing determined by WAXS. However, the studies have compared very different macromolecular chains, and no clear correlations for our materials have been found.

#### 3.3.6. Differential Scanning Calorimetry, DSC, and Thermogravimetric Analysis (TGA)

The results of glass transition temperatures, Tg, degradation temperatures, Td, and char yields for polyimides and copolyimides using the DSC and TGA techniques are detailed in Table 2.

As we mentioned before, it was observed that the aromatic polyimides obtained in this work presented high glass transition temperatures, Tg, with values larger than 310 °C. The highest corresponded to BPDA-6F (323 °C), and the lowest one to iBPDA-6F (313 °C). This trend may be due to the different packing of the macromolecular chains.

**Figure 7:**
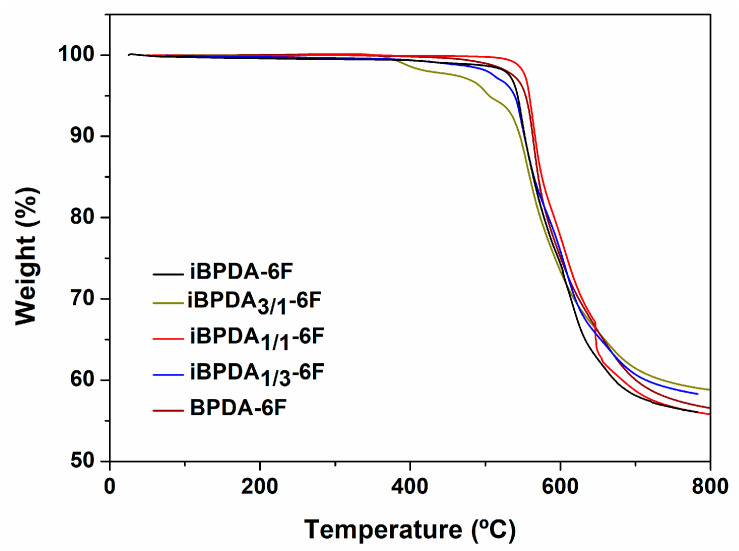
TGA thermograms of heat-treated films.

**Figure 8:**
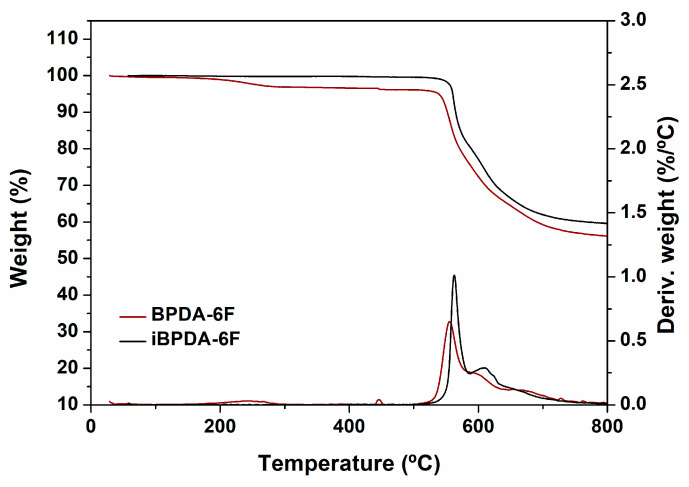
TGA thermograms of polyimide films.

Thermogravimetric analysis results are shown in Figure 7 and Figure 8, Figures S23 and S24 of the supporting information section.

The TGA results indicated that all polymer materials possess excellent thermal stability, with initial decomposition temperatures above 500 °C, regardless of the dianhydride analyzed. The high values seem to correspond to the presence of aromatic, heterocyclic systems, and C-F bonds.

As expected, the char yields were very high, which can be explained by the aromatic character of these polyimides, and were quite similar in all cases. For the BPDA-derived homopolymer, the values coincided with those described in the literature [60].

#### 3.3.7. Mechanical Properties

Mechanical properties are displayed in Table S5 (supporting information, Section S9).

**Figure 9:**
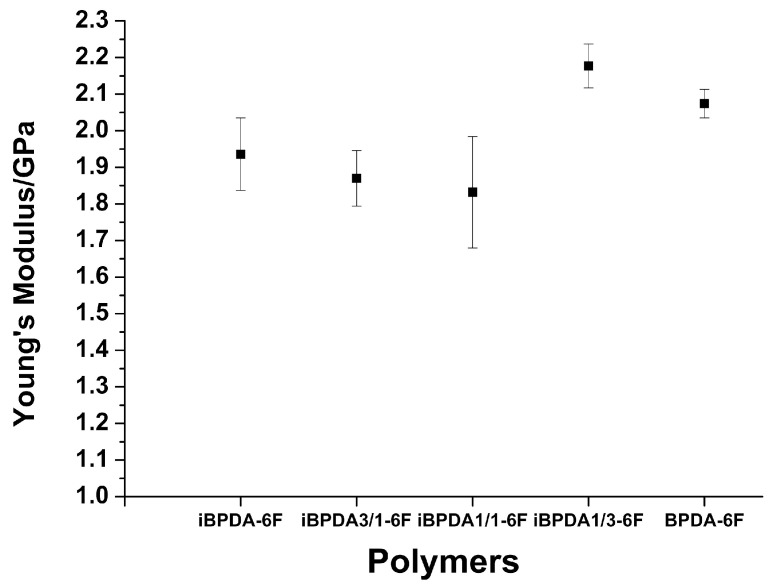
Young’s moduli of polyimides.

Tensile strength and Young’s modulus values were high in all cases. In addition, the elongation at break increased with the proportion of BPDA. The comparison of Young’s modulus between the different polymers is visualized in Figure 9, which shows that the homopolymers, with greater structural regularity, showed higher values than the copolymers, where the random distribution of the units of both dianhydrides must affect the regularity of the packing and, therefore, their modulus value.

The good mechanical properties allow for their use in high-pressure gas purification applications.

#### 3.3.8. Dynamomechanical Properties

**Figure 10:**
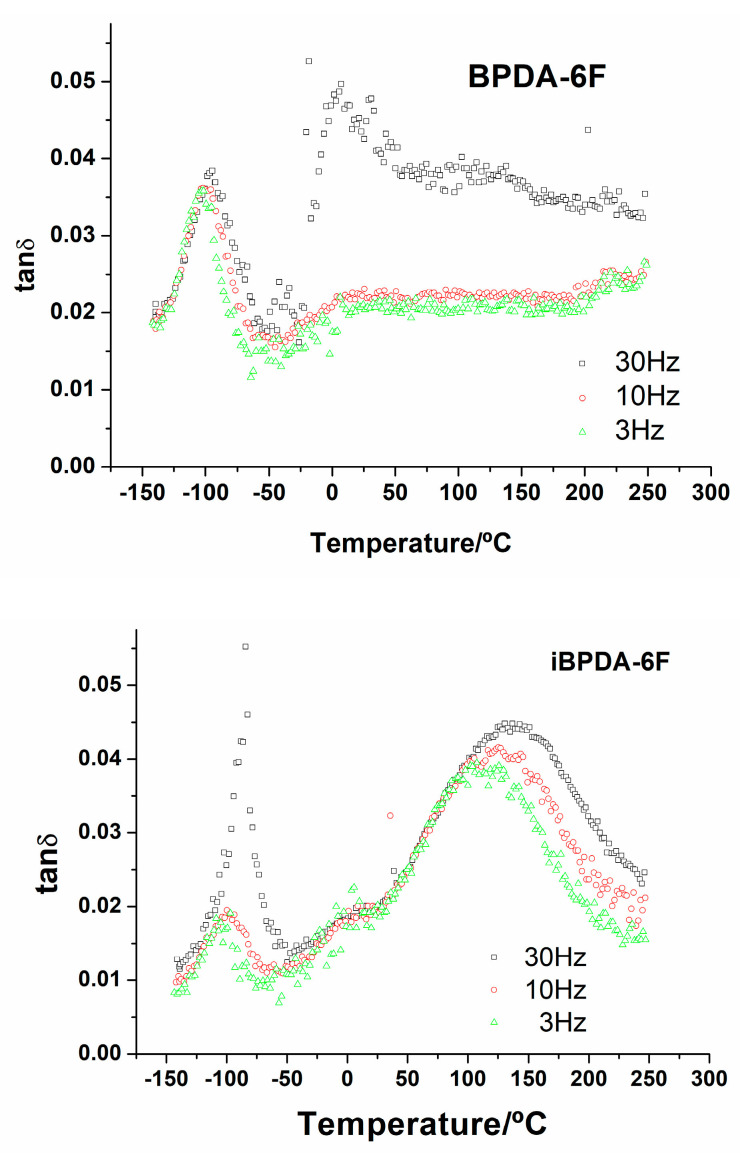
DMTA results (tang δ vs. T) for BPDA-6F (**top**) and iBPDA-6F (**bottom**).

Figure 10 shows tang δ as a function of temperature for iBPDA-6F and BPDA-6F (the DMTA graphs for copolymers are depicted in the supporting information section, Section S10).

It can be observed that both homopolymers and copolymers showed a relaxation process in the low-temperature zone (peak I), which shifted to higher temperatures with the operation frequency. In the higher temperature zone, the polyimide BPDA-6F showed another relaxation process (peak II), demonstrating the same behavior between temperature and frequency. This relaxation process was also detected in the copolymers. However, as the iBPDA comonomer content increased, its intensity decreased, making it difficult to detect the maximum of the peak for some of them. All polymers showed a small shoulder around 0 °C. The apparent activation energy was determined from an Arrhenius representation, taking the temperature of the peak maximum at a determined frequency. Table S6, SI gives the temperature values at 10 Hz and the activation energies of both processes.

**Figure 11:**
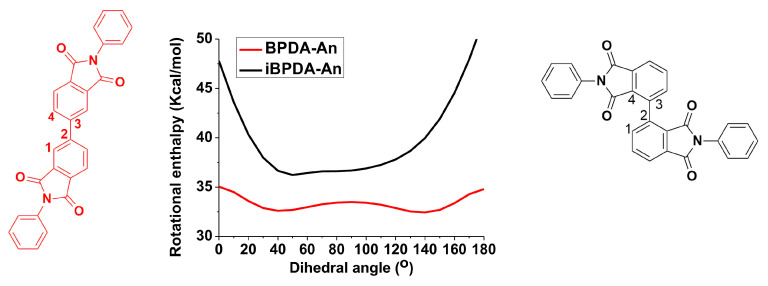
Semiempirical AM1 modeling [47,61] of the rotational enthalpy barrier, of iBPDA-An and BPDA-An models, through the 1-2-3-4dihedral angle.

Both relaxations exhibited an Arrhenius-like behavior characteristic of secondary relaxations, generally associated with local motions of the molecular chain. The high activation energy values, E_a_, further suggest a certain cooperative character of these motions [46,60]. However, no clear dependence of E_a_ on iBPDA content was observed in the copolymers. The relaxation process associated with peak I could be related to the different structural unit provided by the dianhydride used. The iBPDA-derived homopolyimide has a more rigid molecular structure than that corresponding to BPDA due to a very high rotational energy barrier at the CAr-CAr’ (1-2-3-4 dihedral angle) bond of the biphenyl moiety (Figure 11). For this reason, the activation energy that iBPDA-6F must overcome to permit local motions is high.

The data obtained did not allow us to relate the relaxation process associated with peak II with any type of intramolecular or intermolecular motion.

### 3.4. Gas Separation

Table 3, Tables S7 and S8 of the supporting information section show the permeability, diffusivity, and solubility results for the different membranes as well as the ideal selectivities and diffusivity selectivities.

**Figure 12:**
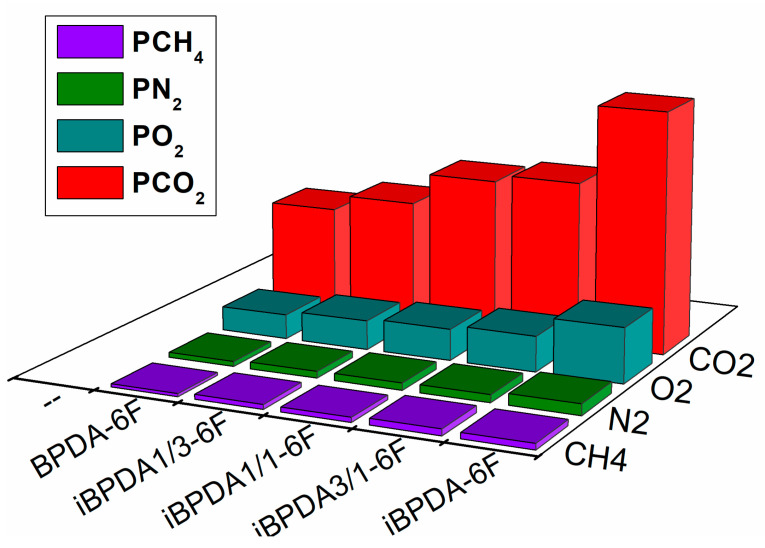
Permeability values of polymers.

**Figure 13:**
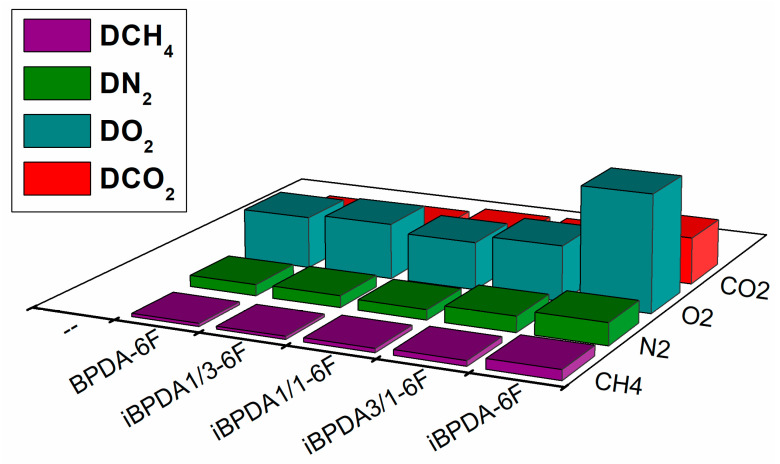
Comparison of gas diffusivity values of polymers.

**Figure 14:**
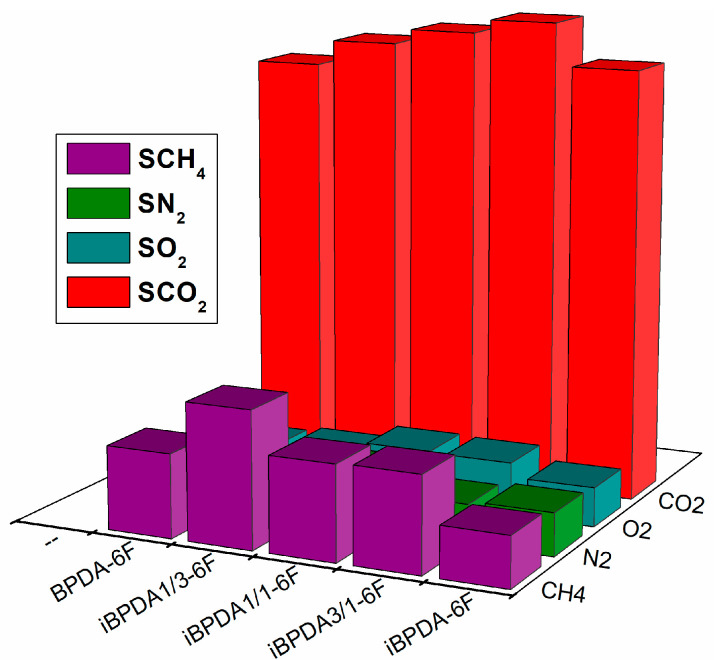
Comparison of gas solubility values of polymers.

**Figure 15:**
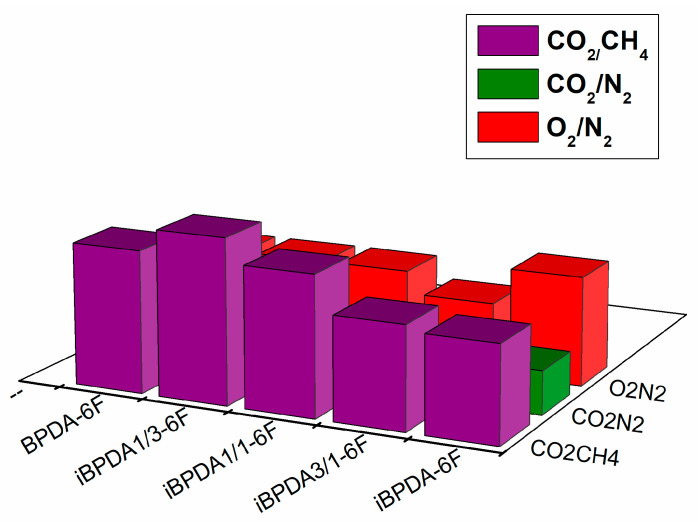
Permeability vs. selectivity.

For the better visualization and discussion of the results obtained in the gas separation measurements, the values of the tables have been graphically represented (Figure 12, Figure 13, Figure 14 and Figure 15).

It was observed that the homopolyimide iBPDA-6F showed the best permeability values for all the gases studied. When comparing iBPDA-6F with its homopolyimide isomer BPDA-6F, it could be observed that the permeability enhancement was between 92 and 126%, depending on the gas studied. Furthermore, when the permeability was compared with the mole fraction of the iBPDA component a linear increase was observed, except for the BPDA-6F polymer, which presents a lower value than expected, and for iBPDA-6F, whose permeability was higher than expected.

For gas diffusivity, a parameter that depends directly on the size of the penetrating gas (kinetic diameter), it was observed that the values for polymers having the BPDA monomer were quite similar; deviations lower than 40% iBPDA-6F showed a much higher diffusivity parameter. Therefore, and according to the solubility data discussed in the following section, it can be considered that the improvement in permeability for iBPDA-6F is practically due to its improvement in the diffusion coefficient. The most probable microscopic explanation is complex, since this polymer showed a lower free volume fraction than the rest of the polymers studied in this work. Therefore, considering the X-ray diffractograms, it could be plausible to consider that the free volume distribution could be the main factor that controls the gas separation properties of these materials.

Regarding gas solubilities, when we compared the two homopolymers, iBPDA-6F and BPDA-6F, it was observed that the values of S are practically the same for the gases with the most ideal behavior (nitrogen and oxygen). For condensable gases (the condensability of a gas is directly related to its critical temperature (Tc) [26,62,63]), CO_2_ solubilities showed similar values. In contrast, the CH_4_ solubility value for the BPDA-6F polymer was 68% higher than that of the iBPDA-6F polymer.

In the case of selectivity coefficients for the CO_2_/CH_4_, O_2_/N_2_, and CO_2_/N_2_ gas pairs, the differences were not significant since their values were very similar, and the results fell within the error associated with the determination of permeability (approx. 10%). For the CO_2_/CH_4_ gas pair, an increase in selectivity for the iBPDA-6F polymer was observed. However, when comparing the whole series for this gas pair, the values obtained were very scattered and it was not possible to offer a coherent explanation that would allow us to figure out some relationship between structure and properties.

Finally, to know the capacity of these new polymers to separate gases effectively a comparison was made with a collection of polymeric membranes, plotting the selectivity of the material for a pair of gases with the permeability of the gas that permeates more easily. The distance from that point (P, α) to the empirical Robeson limit allowed us to decide whether that material was suitable to perform that separation.

**Figure 16:**
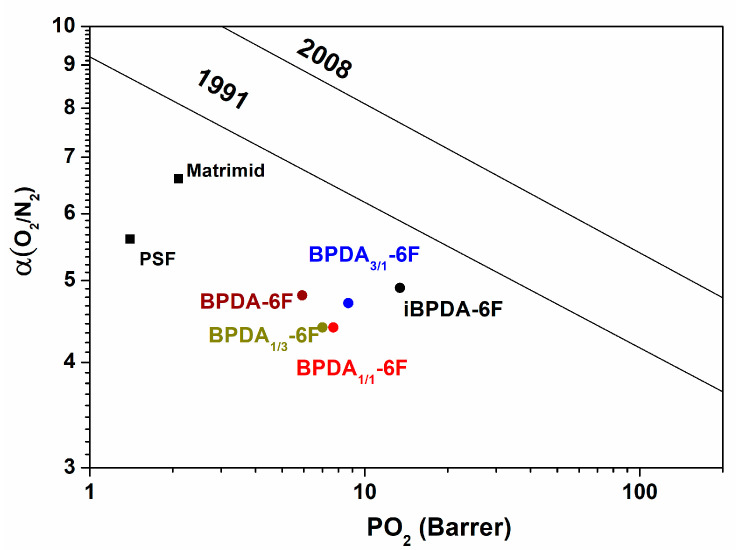
Representation of O_2_ /N_2_ selectivity versus O_2_ permeability.

**Figure 17:**
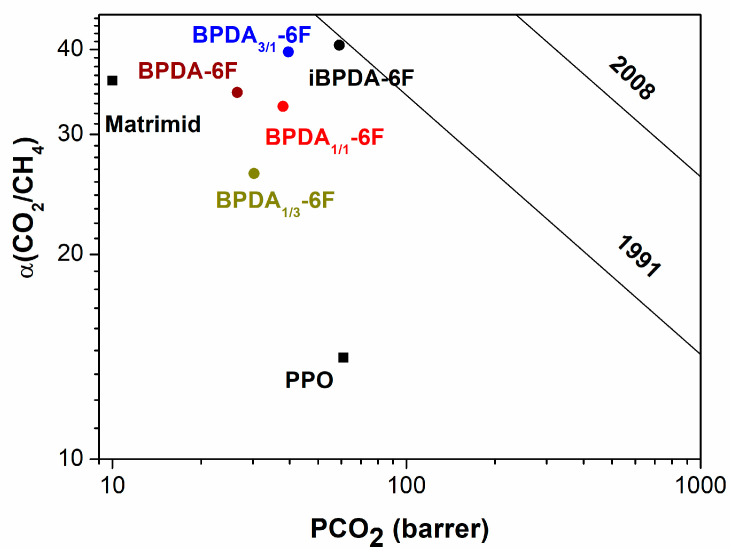
Representation of CO_2_ /CH_4_ selectivity versus CH_4_ permeability.

The polymer materials were compared with PSF, PPO, and Matrimid membranes, which are used industrially in gas purification applications [17,60,64,65]. It was observed in Figure 16 and Figure 17, the O_2_/N_2_ and CO_2_/CH_4_ separation processes, that the polymers of this work, particularly the iBPDA-6F homopolymer, present excellent balances between permeability and selectivity, so they are very close to the 1991 Robeson limit.

Therefore, the polyimides proposed in this work showed interesting gas separation properties. In particular, the iBPDA-6F homopolymer showed good gas separation properties, especially for such an important separation process as the removal of acid gases (CO_2_) in natural gas; the proposed polymers showed application potential.

## 4. Conclusions

The use of molecular modeling techniques has allowed us to design a new family of aromatic polyimides derived from the 2,2′,3,3′-biphenyltetracarboxylic dianhydride (iBPDA), which imparts high chain stiffness and high free volume to the polymers derived from that monomer.

Through a study of the synthetic methods used in the literature to obtain the iBPDA monomer, a new high-yield synthesis route has been optimized to produce this monomer with high purity. As a result, aromatic homopolyimides and copolyimides of high molecular weight were obtained. Moreover, the viscosity values of our polymers were much higher than those obtained by other authors.

The thermal properties of these polymers were very good, with glass transition temperatures higher than 310 °C and degradation onset temperatures, in inert atmosphere, higher than 500 °C.

The properties of these aromatic polyimides were excellent, presenting good solubility in common organic solvents such as chloroform and tetrahydrofuran and high values of Young’s modulus and tensile strength. Thus, it has been possible to manufacture homogeneous high-quality membranes capable of withstanding the pressures required in gas purification.

All the polymers showed an amorphous nature. Additionally, the average spacing of iBPDA-6F homopolyimide showed a d-spacing of 6.6 Ǻ, which is higher than the average diffraction spacing of BPDA-6F, 5.7 Ǻ.

Fractional free volumes were calculated from the density values, which presented values close to one of the best polyimides used in this field, 6FDA-6FpDA. The BPDA-6F polymer presented a higher FFV value, 0.192, than that of iBPDA-6F, 0.186.

The permeation properties of these membranes were very interesting, presenting high permeability values to all gases used together with correct selectivity values. In all cases, the iBPDA-6F membrane presented the best values for gas productivity. Furthermore, it was found that the diffusivity coefficient mainly controls the permeation properties.

Contrary to what is accepted in the theory of gas diffusion in polymeric membranes, the iBPDA-6F polymer, even presenting a lower value of free volume, has better separation values than the rest of the series. The most coherent explanation for this fact is to consider that the free volume distribution of this structure, due to the highly rigid and contorted nature of the iBPDA monomer, is less dispersed than that of the polymers incorporating BPDA.

A comparison of these polymers against a variety of commercial polymers and polymers synthesized in our laboratory, which possess excellent separation values, allows us to ensure that these polymers fall in the commercial separation zone and are very close to the 1991 Robeson limit. Remarkably, the iBPDA-6F polymer presented an outstanding CO_2_/CH_4_ gas pair separation ability, which could allow these structures to be used in industrial processes thanks to the appropriate combination of thermal stability, good processability, and excellent separation properties.

## Figures and Tables

**Table 1:** Polymer Acronyms.

iBPDA (mol)	BPDA (mol)	6FpDA (mol)	Acronym
1.0	0	1.0	iBPDA-6F
0.75	0.25	1.0	iBPDA_3/1_-6F
0.5	0.5	1.0	iBPDA_1/1_-6F
0.25	0.75	1.0	iBPDA_1/3_-6F
0	1.0	1.0	BPDA-6F

**Table 2:** Thermal Properties of Polyimides.

Polymer	Tg/°C	Td/°C	Char yield at 800 °C (%)
iBPDA-6F	313	510	58
iBPDA_3/1_-6F	315	510	58
iBPDA_1/1_-6F	316	500	57
iBPDA_1/3_-6F	318	500	58
BPDA-6F	323	515	58

**Table 3:** Permeability values, P (barrers).

Polymer	PO_2_	PN_2_	PCH_4_	PCO_2_
iBPDA-6F	13.42	2.76	1.46	59.07
iBPDA3/1-6F	8.71	1.87	1.60	39.69
iBPDA1/1-6F	7.68	1.75	1.15	38.02
iBPDA1/3-6F	7.01	1.58	1.15	30.29
BPDA-6F	5.92	1.24	0.76	26.55

## Data Availability

Not applicable.

## Supplementary Materials

The following supporting information can be downloaded at: https://www.mdpi.com/article/10.3390/polym15061333/s1, S1. Monomer synthesis, S2. Polymer synthesis, S3. Elemental microanalysis data, S4. Infrared spectra (FTIR) of polymers, S5. Viscosities and molecular weights, S6. Solubilities, S7. Densities and fractional free volumes, S8. Thermal properties of polyimides; differential scanning calorimetry (DSC), and thermogravimetric analysis (TGA), S9. Mechanical properties, S10. Dynamomechanical properties (DMTA), S11. Activation energy, S12. Gas permeability, diffusivity, and selectivity values of membranes, S13. Error calculations.
