# Supplementary material for: Isomeric Aromatic Polyimides Containing Biphenyl Moieties for Gas Separation Applications
Source: Polymers (Basel). 2023 Mar 7;15(6):1333. doi: 10.3390/polym15061333 (PMC10056342; doi:10.3390/polym15061333)
Supplement: Supplementary file 1 [file polymers-15-01333-s001.zip › polymers-2218874-supplementary.pdf]

Supporting information for

Isomeric aromatic polyimides containing biphenyl moieties for gas separation applications.

Laura Matesanz-Niño[1,2,3], David Cuellas[1], Carla Aguilar-Lugo[4], Laura Palacio[2], Alfonso González-Ortega[3], José G de la Campa[1], Cristina Álvarez[1,2], Ángel E Lozano[1,2,5,*].

1. Department of Macromolecular Chemistry, Institute of Polymer Science and Technology, ICTP-CSIC, Juan de la Cierva 3, E-28006 Madrid, Spain.
2. SMAP, UA-UVA_CSIC, Research Unit associated to CSIC. Faculty of Science, University of Valladolid, Paseo Belén 11, E-47011 Valladolid, Spain.
3. Department of Organic Chemistry, Faculty of Sciences, University of Valladolid, Paseo Belén 7, E-47011 Valladolid, Spain.
4. Faculty of Chemistry, National Autonomous University of Mexico, Cd. University, Coyoacán, C. P. 04510 México, CDMX, México.
5. UI CINQUIMA, University of Valladolid, Paseo Belén 5, E-47011 Valladolid, Spain.
*: corresponding author, lozano@ictp.csic.es

S1. Monomer synthesis

S1.1. Preparation of 2,2′,3,3′-tetramethylbiphenyl (iBPTM) (Scheme S1, Figures S1-S3).

S1.2. Preparation of 2,2′,3,3′-biphenyltetracarboxylic acid (iBPTA) (Scheme S2, Figures S4-S6).

S1.3. Preparation of 2,2′,3,3′-biphenyltetracarboxylic dianhydride (iBPDA) (Scheme S3, Figures S7-S9).

S2. Polymer synthesis

S2.1. ^1H, ^{13}C, HSQC-NMR for iBPDA-6F homopolyimide (Figures S10-S12).

S2.2. ^1H, ^{13}C, HSQC-NMR for BPDA-6F homopolyimide (Figures S13-S15).

S2.3. ^1H, ^{13}C-NMR for $BPDA_{3/1}$-6F copolymer (Figures S16, S17).

S2.4. ^1H, ^{13}C-NMR for $BPDA_{1/1}$-6F copolymer (Figures S18, S19).

S2.5. ^{1}H, ^{13}C-NMR for BPDA$_{1/3}$-6F copolymer (Figures S20, S21).

S3. Elemental microanalysis data (Table S1)

S4. Infrared spectra (FTIR) of polymers (Figure S22)

S5. Viscosities and molecular weights (Table S2)

S6. Solubilities (Table S3)

S7. Densities and fractional free volumes (Table S4)

S8. Thermal properties of polyimides; differential scanning calorimetry (DSC), and thermogravimetric analysis (TGA) (Figures S23, S24)

S9. Mechanical properties (Table S5)

S10. Dynamomechanical properties (DMTA) (Figures S25-27)

S11. Activation energy (Table S6)

S12. Gas permeability, diffusivity, and selectivity values of membranes (Table S7, S8)

S13. Error calculations

S1. Monomer synthesis

S1.1. Preparation of 2,2′,3,3′-tetramethylbiphenyl (iBPTM).

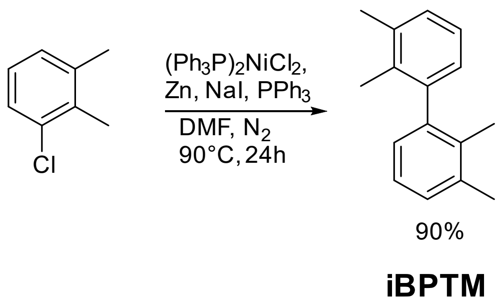

Scheme S1. *Synthesis of 2,2′,3,3′-tetramethylbiphenyl.*

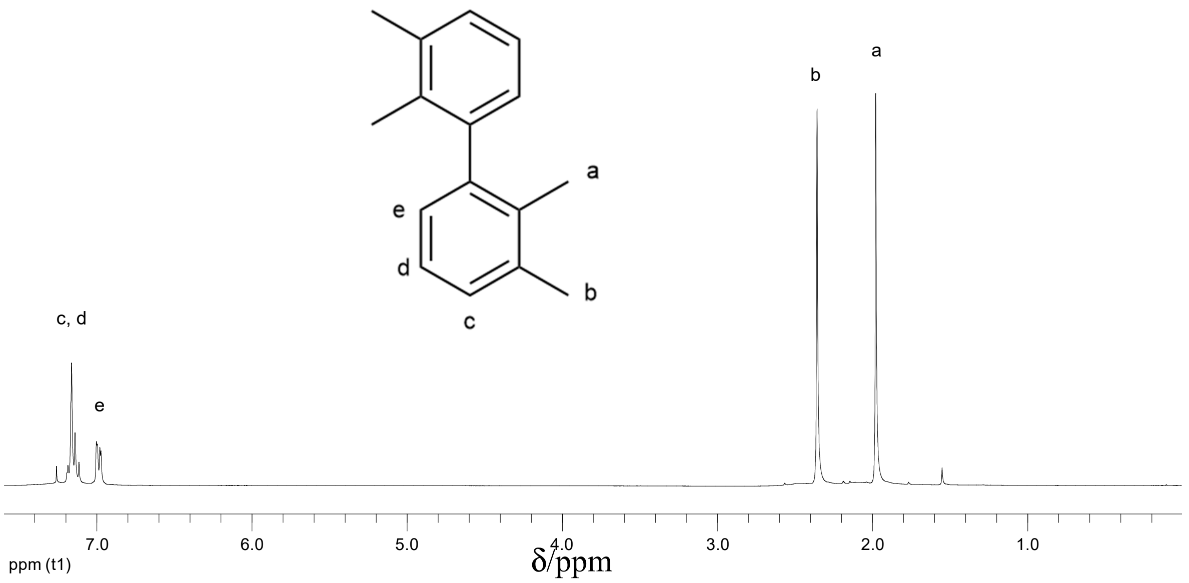

Figure S1. *^{1}H-NMR for 2,2′, 3,3′-tetramethylbiphenyl.*

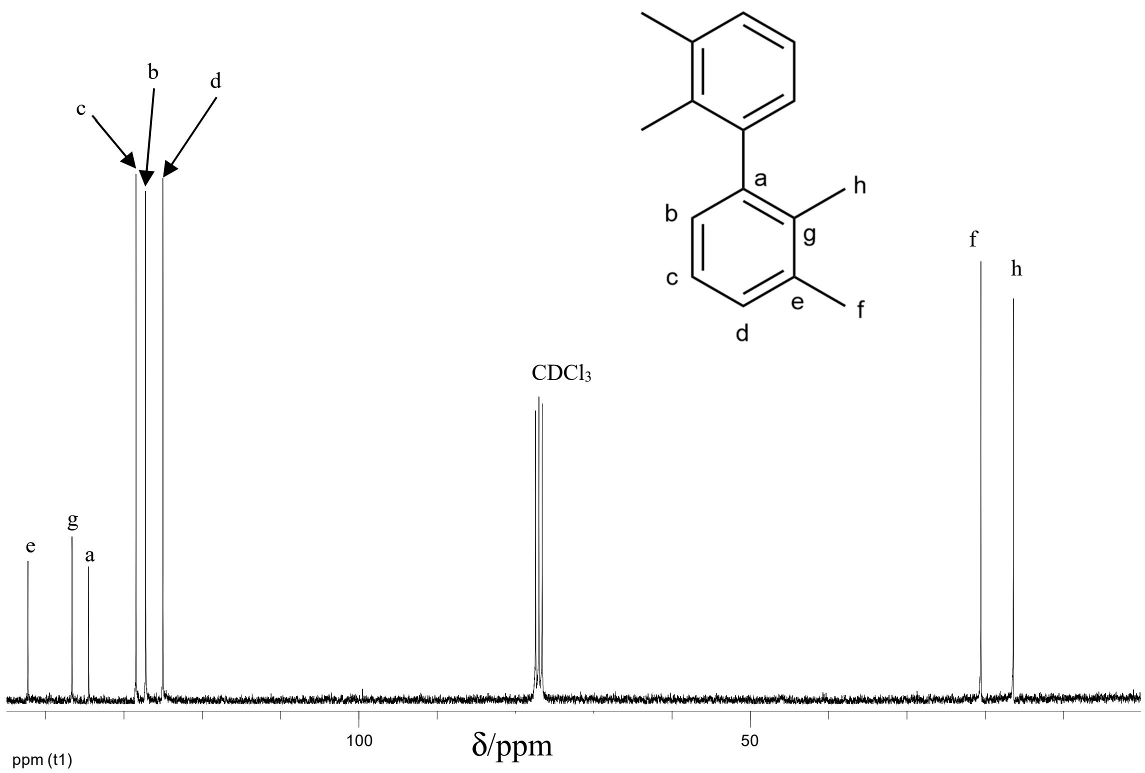

***Figure S2**. ^{13}C-NMR for 2,2′, 3,3′-tetramethylbiphenyl.*

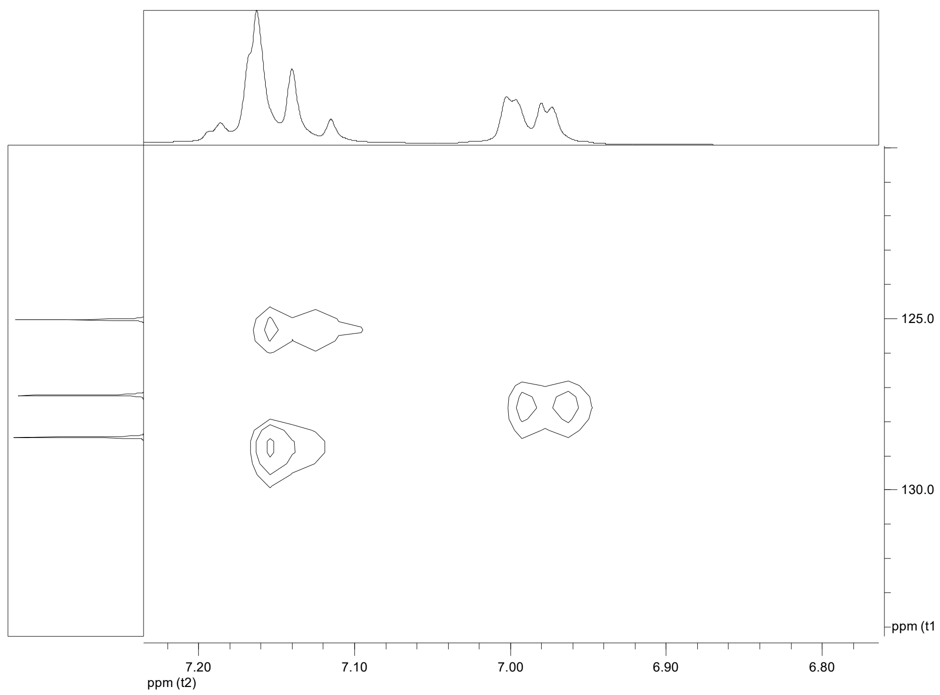

***Figure S3**. HSQC-NMR for 2,2′, 3,3′-tetramethylbiphenyl.*

S1.2. Preparation of 2,2′, 3,3′-biphenyltetracarboxylic acid (iBPTA).

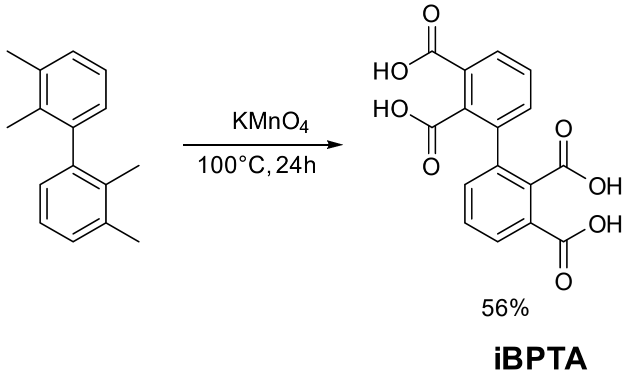

Scheme S2. *Synthesis of 2,2′, 3,3′-biphenyltetracarboxylic acid.*

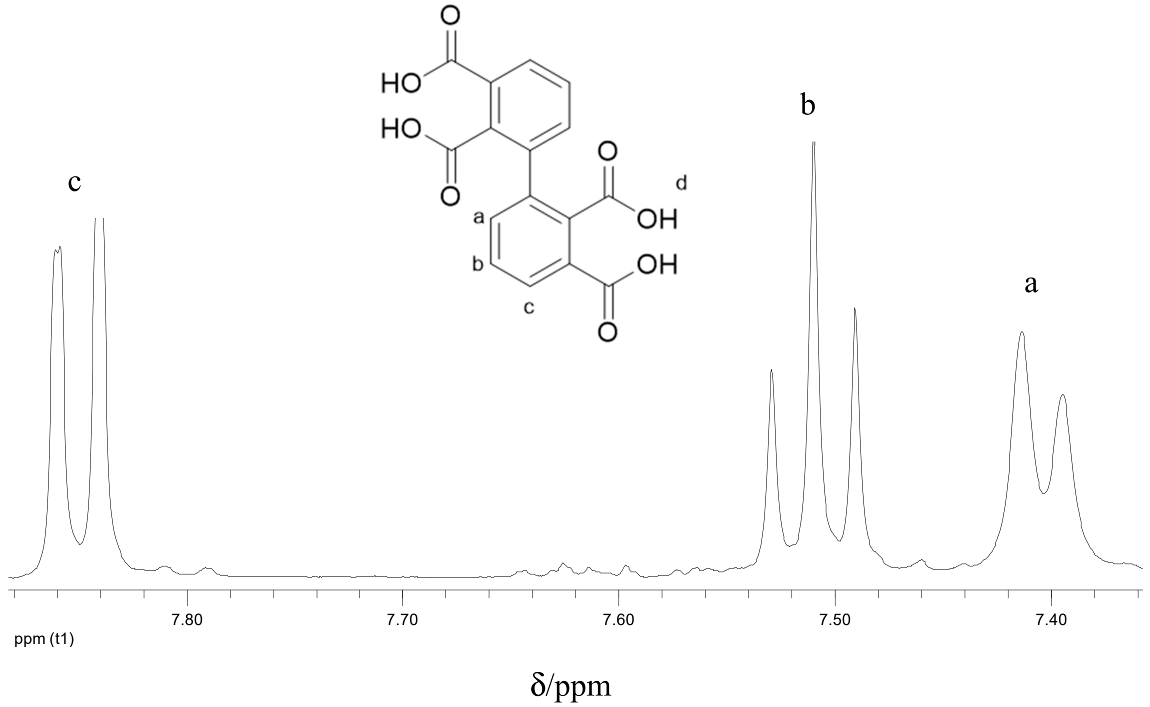

Figure S4*. ^{1}H-NMR for 2,2′, 3,3′-biphenyltetracarboxylic acid*

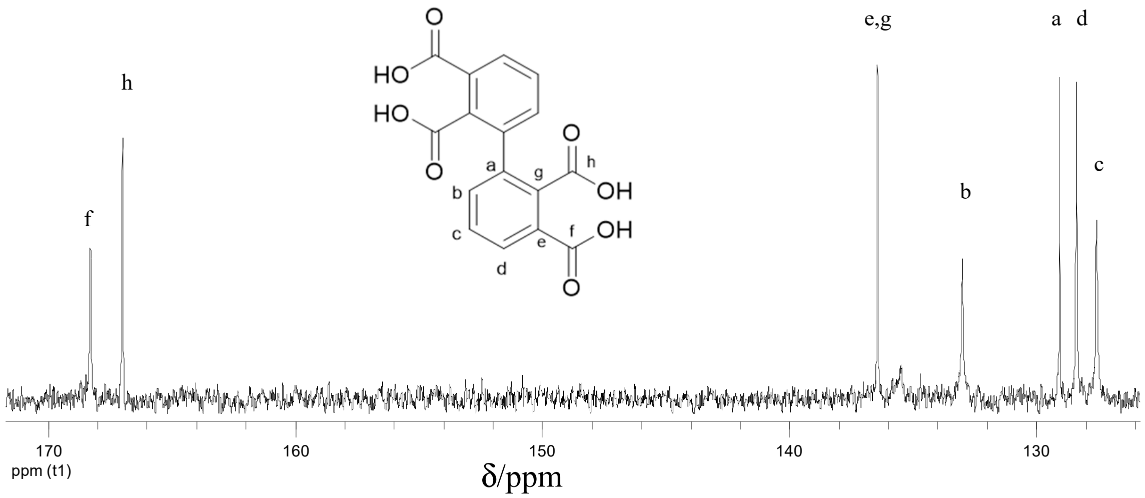

Figure S5. *^{13}C-NMR for 2,2′, 3,3′-biphenyltetracarboxylic acid.*

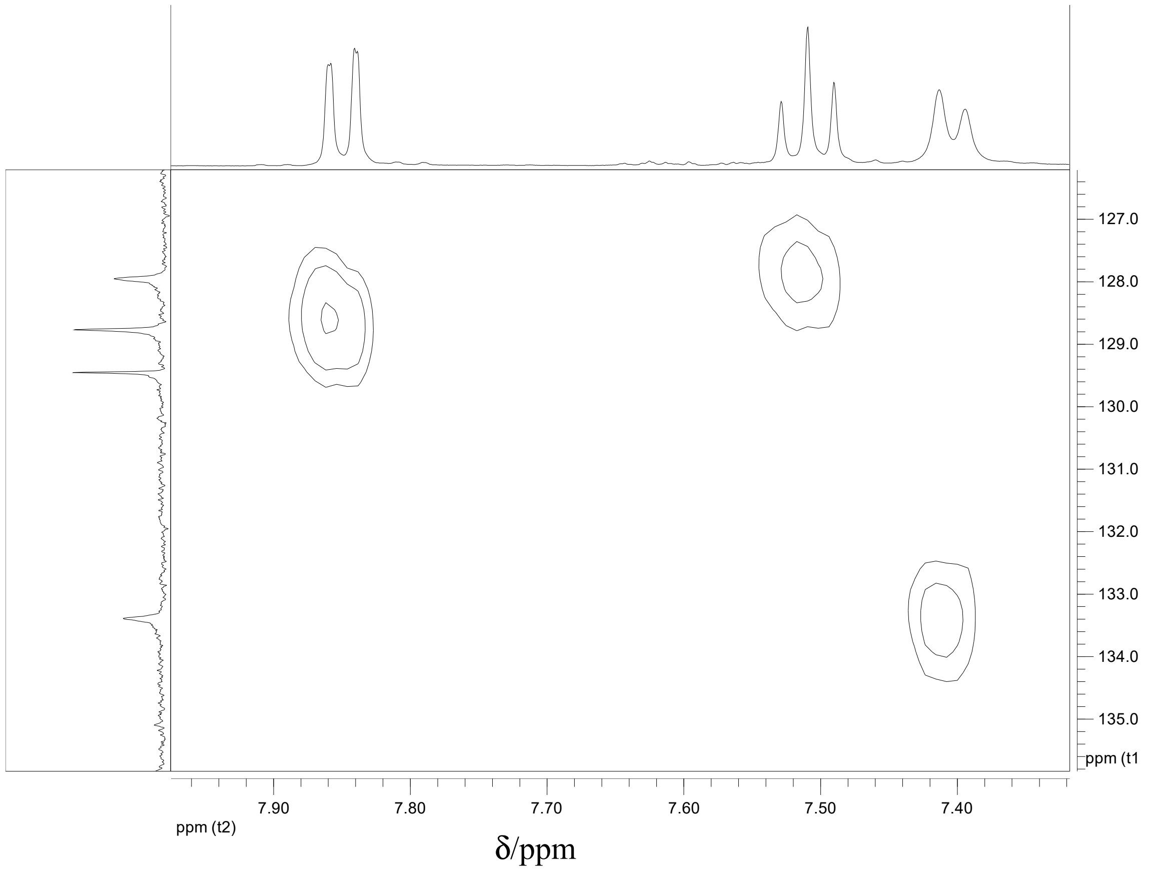

Figure S6*. HSQC- NMR for 2,2′, 3,3′-biphenyltetracarboxylic acid.*

S1.3. Preparation of 2,2′,3,3′-biphenyltetracarboxylic dianhydride (iBPDA).

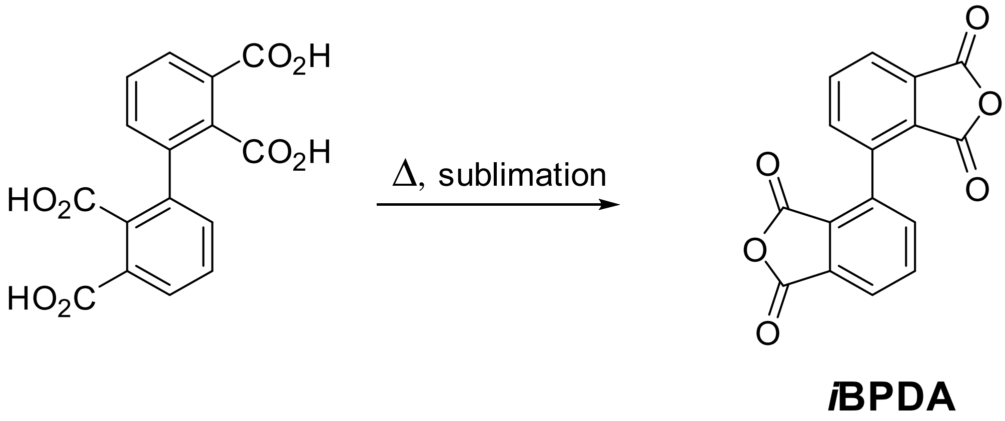

Scheme S3. *Synthesis of iBPDA.*

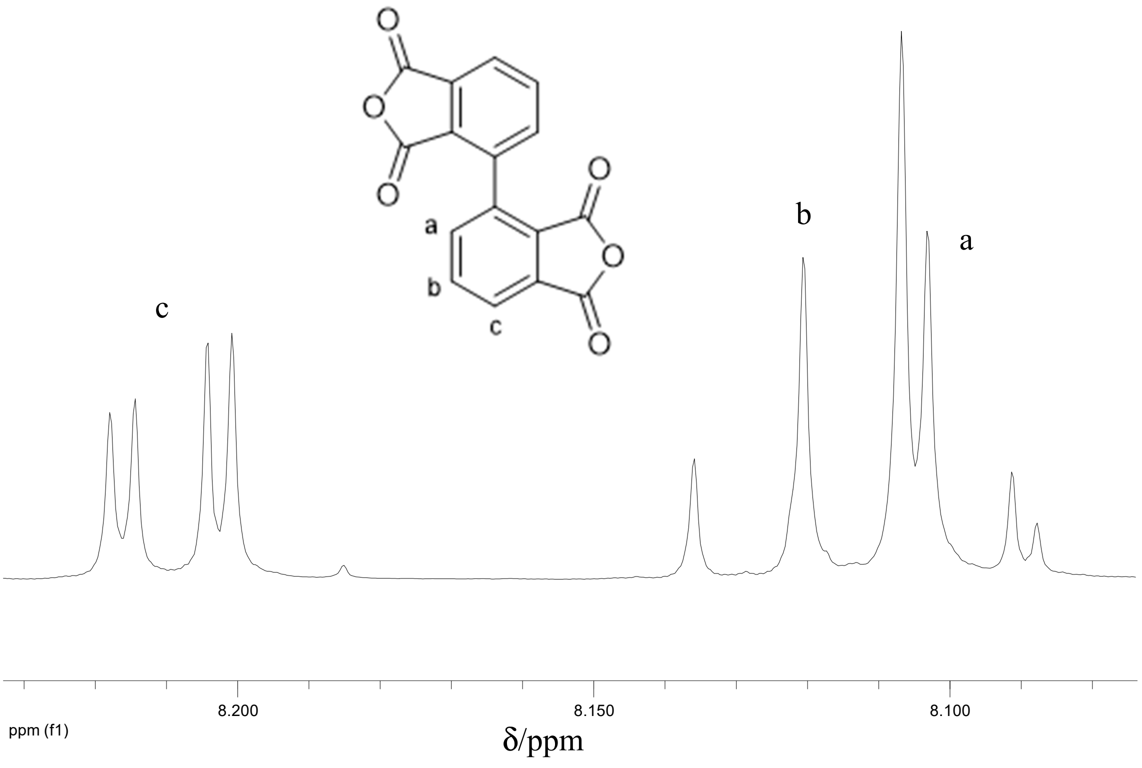

Figure S7. *^{1}H- NMR for iBPDA.*

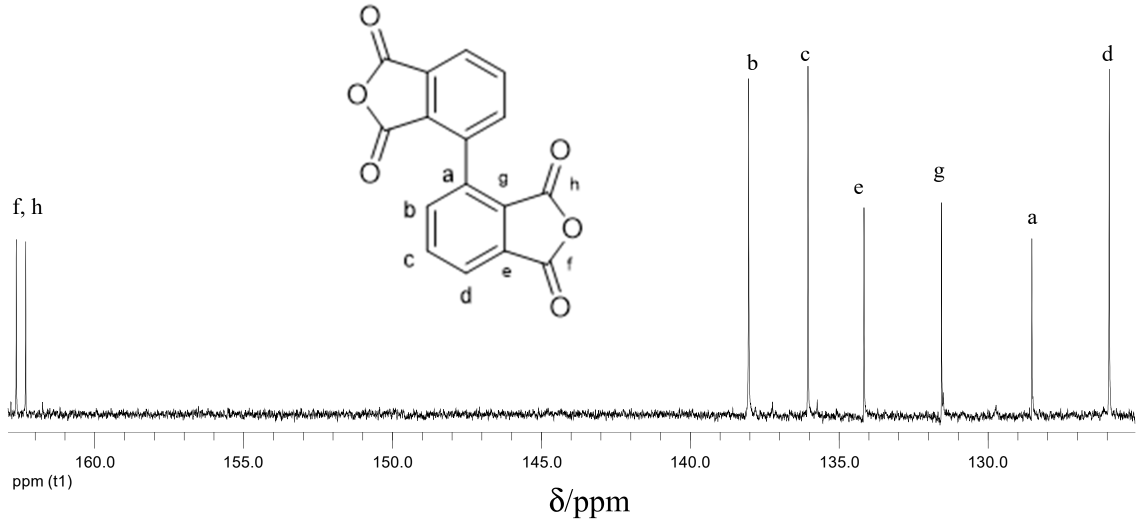

***Figure S8**. ^{13}C- NMR for iBPDA.*

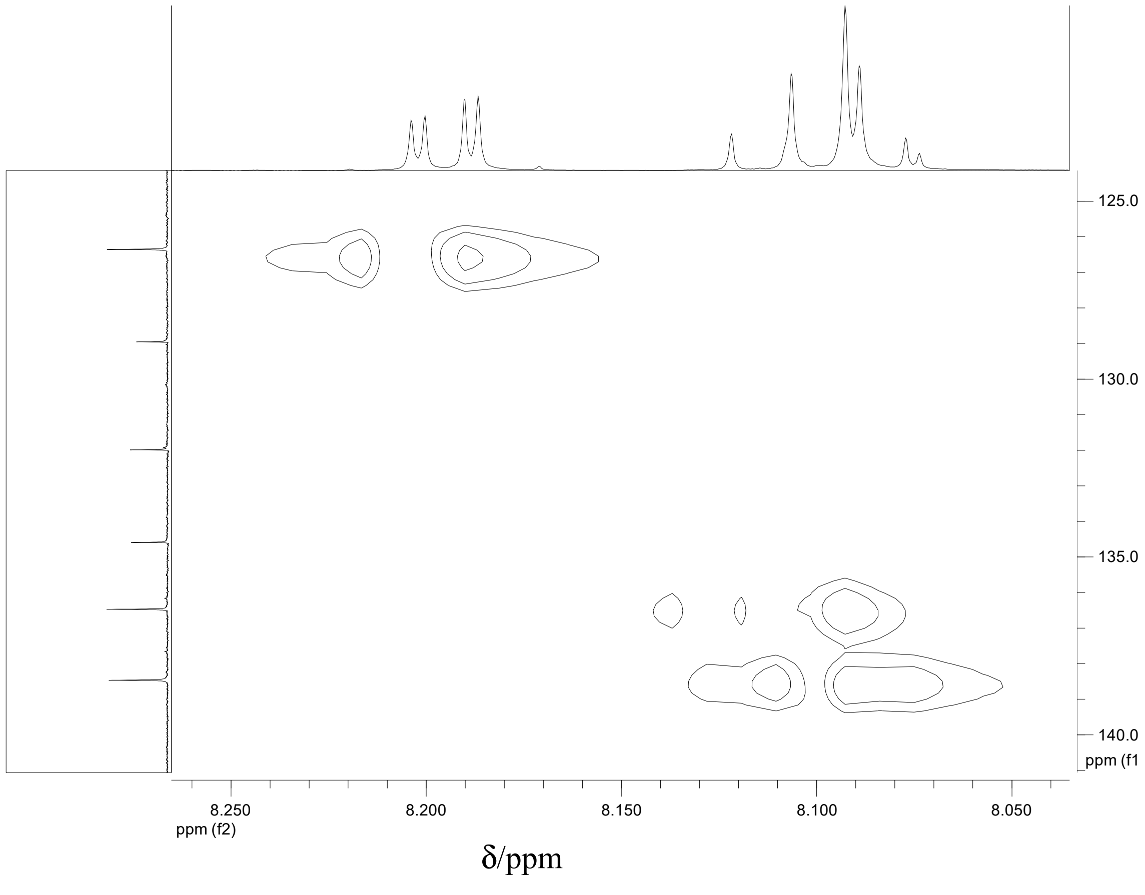

***Figure S9**. HSQC- NMR for iBPDA.*

S2. Polymer synthesis

S2.1. ^{1}H, ^{13}C and HSQC-NMR of iBPDA-6F homopolyimide

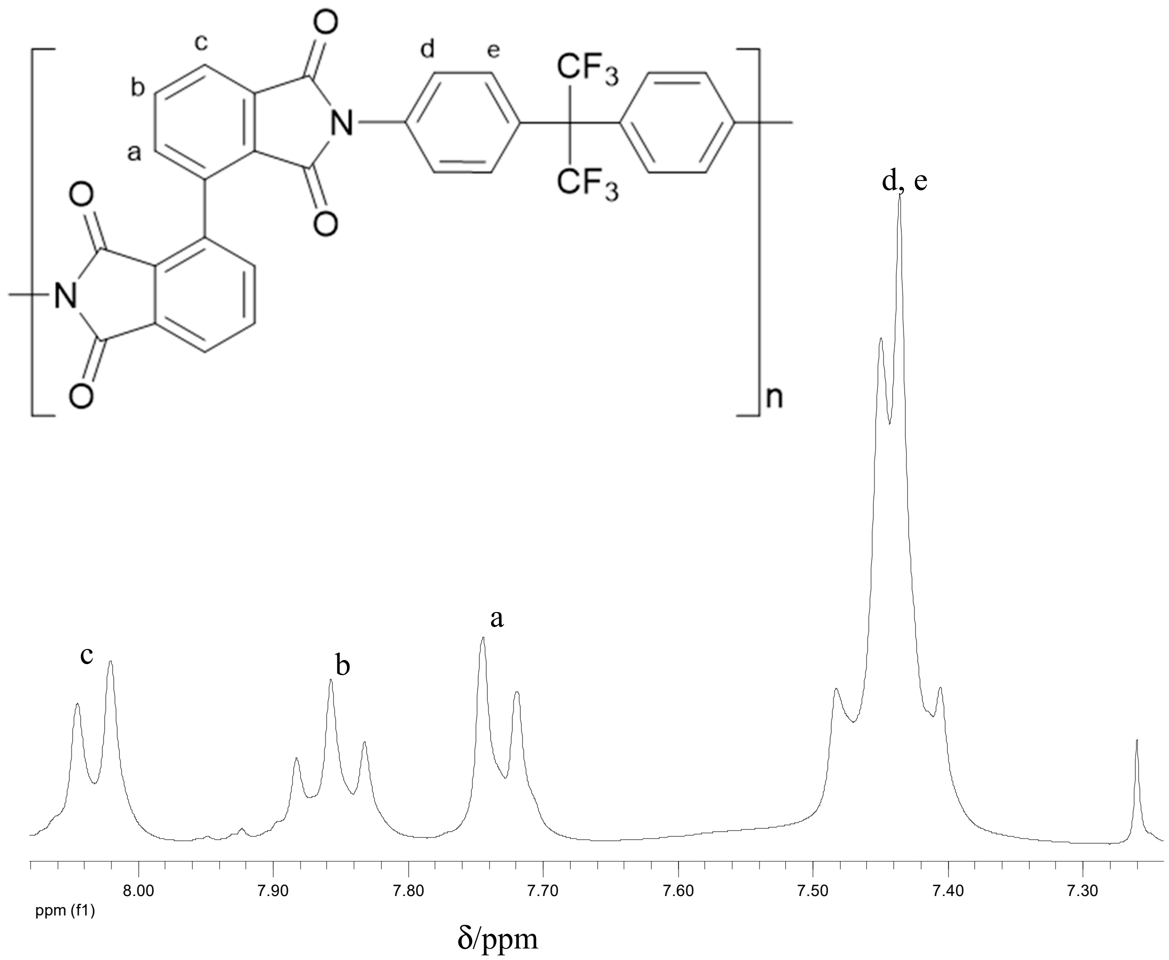

Figure S10. *^{1}H- NMR for iBPDA-6F homopolyimide.*

^{1}H- NMR, 300 MHz, $CDCl_3$: 8.03 (2H, dd, 2H_c, $J_{H_c\text{-}H_b}$=7.5 Hz), 7.86 (2H, t, 2H_b, $J_{H_b\text{-}H_a}$=$J_{H_b\text{-}H_c}$=7.5 Hz), 7.73 (2H, d, 2H_a, $J_{H_b\text{-}H_a}$=7.8 Hz), 7.46 (d, 4H, H_e, $J_{H_d\text{-}H_e}$=9.2 Hz), 7.42 (d, 4H, H_d).

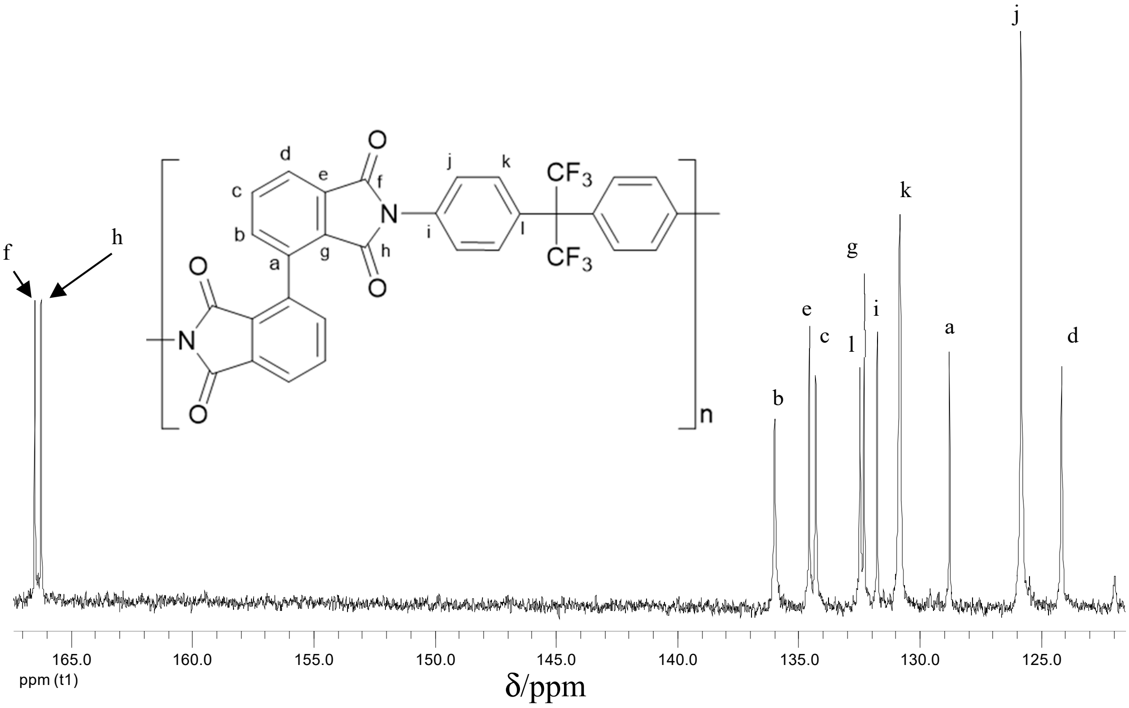

Figure S11. *^{13}C- NMR for the homopolyimide iBPDA-6F.*

^{13}C- NMR, 75 MHz, $CDCl_3$: 166.5 (C_f), 166.2 (C_h), 136.0(C_b), 134.6 (C_e), 134.3 (C_c), 132.5 (C_l), 132.3 (C_g), 131.8 (C_i), 130.8 (C_k), 128.8 (C_a), 125.8 (C_j), 124.2 (C_d).

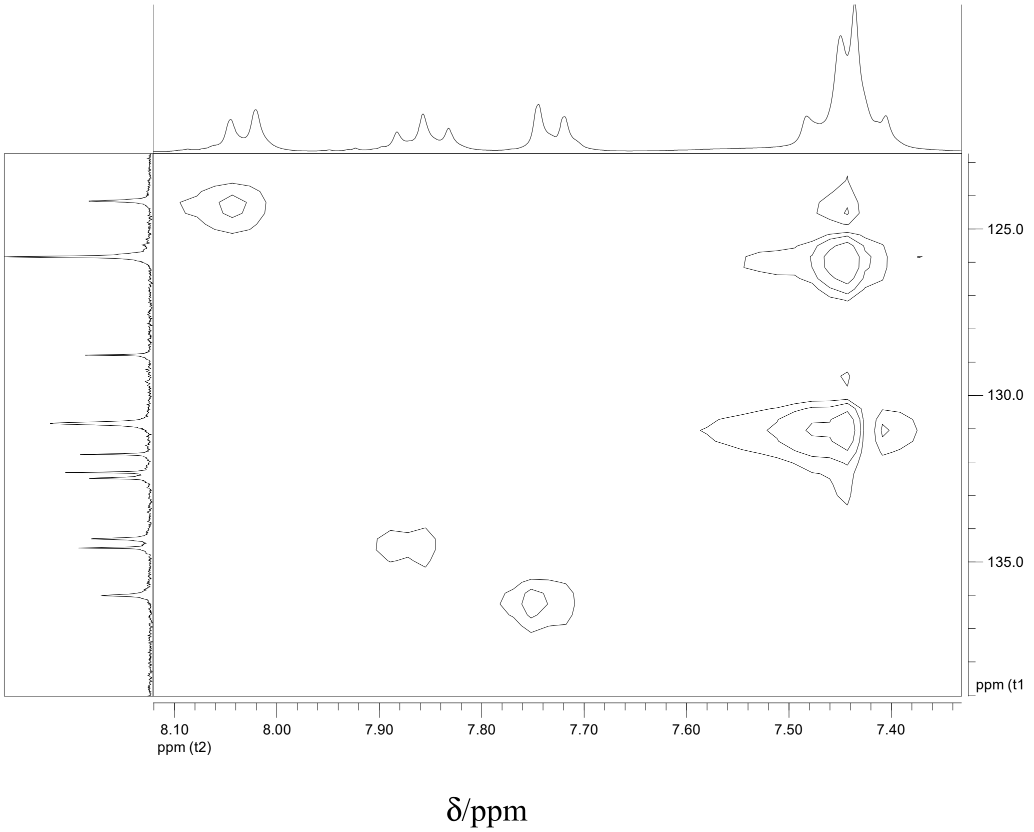

***Figure S12**. HSQC- NMR for iBPDA-6F homopolyimide.*

S2.2. ^{1}H, ^{13}C, HSQC-NMR for BPDA-6F homopolyimide

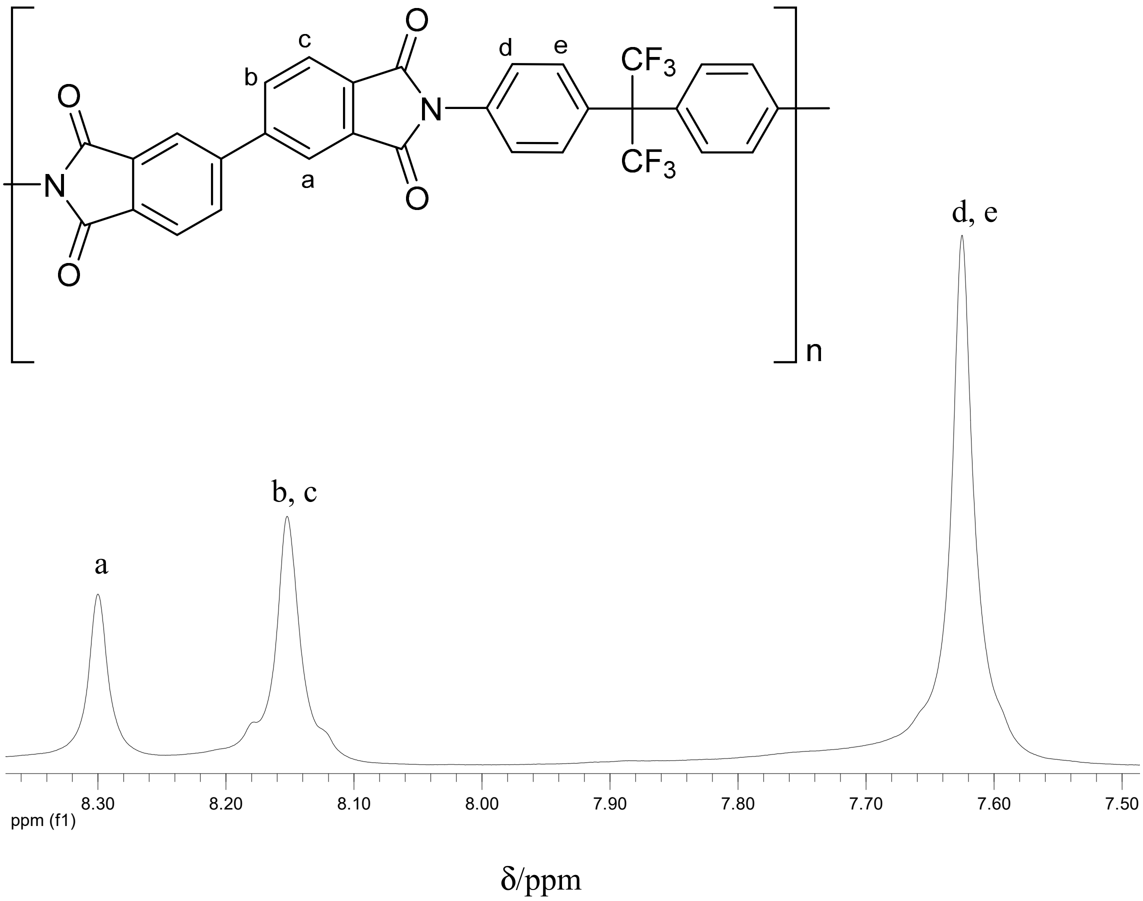

Figure S13. *^{1}H-NMR for homopolyimide BPDA-6F.*

^{1}H-NMR, 300 MHz, $CDCl_3$: 8.3 (s, 2H, 2H_a), 8.2-8.1 (m, 4H, H_c, H_b,), 7.6 (m, 8H, H_d, H_e).

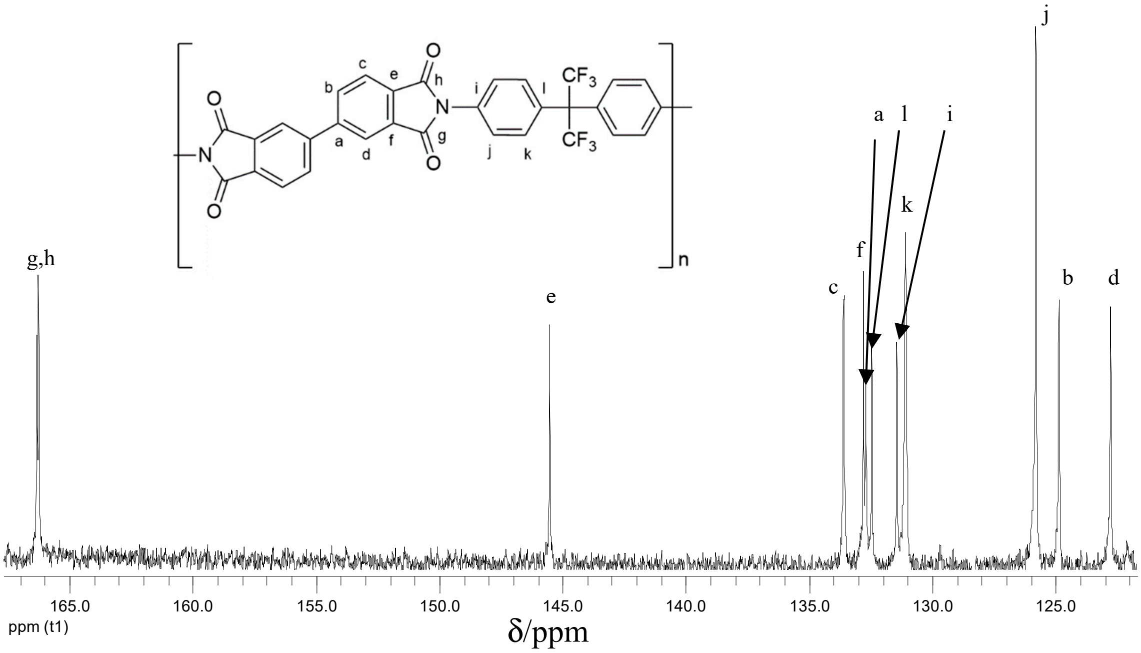

Figure S14. *^{13}C- NMR for homopolyimide BPDA-6F.*

^{13}C- NMR, 75 MHz, $CDCl_3$: 166.3 (C_g), 166.2 (C_h), 145.5 (C_e), 133.6 (C_c), 132.7 (C_a), 132.5 (C_l), 131.4 (C_i), 131.1 (C_k), 125.8 (C_j), 124.9 (C_b), 122.8 (C_d).

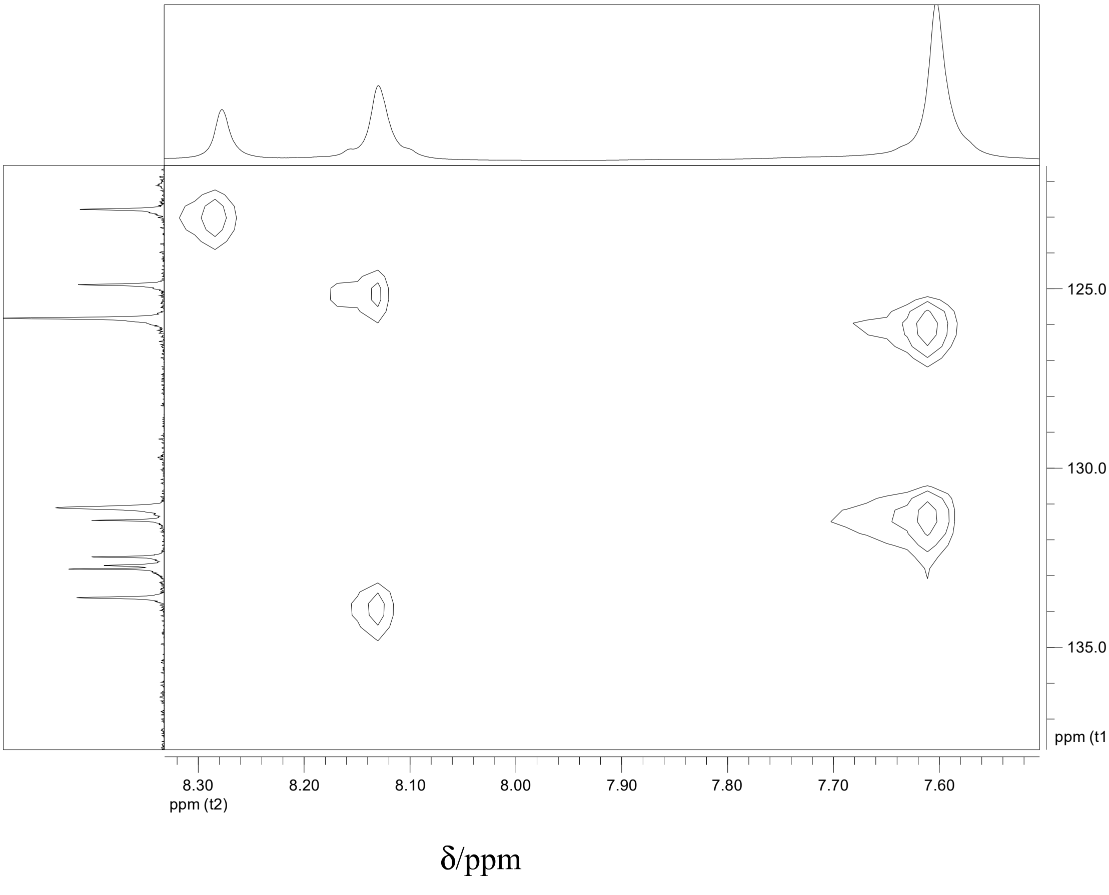

Figure S15. *HSQC- NMR for BPDA-6F homopolyimide.*

S2.3. ¹H, ¹³C-NMR for BPDA$_{3/1}$-6F copolymer

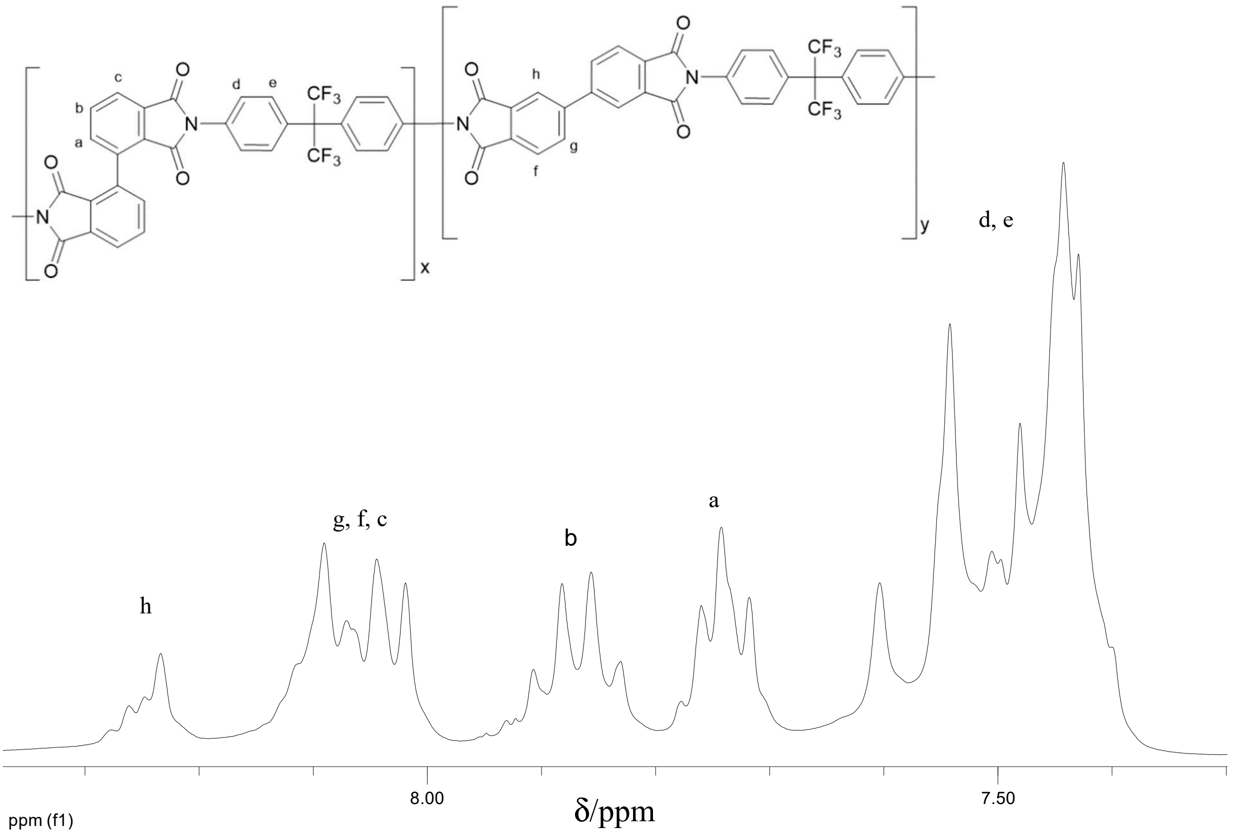

***Figure S16.** ¹H-NMR for BPDA$_{3/1}$-6F copolymer.*

¹H- NMR, 300 MHz, $CDCl_3$: 8.30-8.20 (m, 2H, H_h), 8.15-7.97 (m, 10H, H_g-H_f-H_c), 7.95-7.80 (m, 6H, H_b), 7.79-7.68 (m, 6H, H_a), 7.67-7.35 (m, 32H, H_d, H_e).

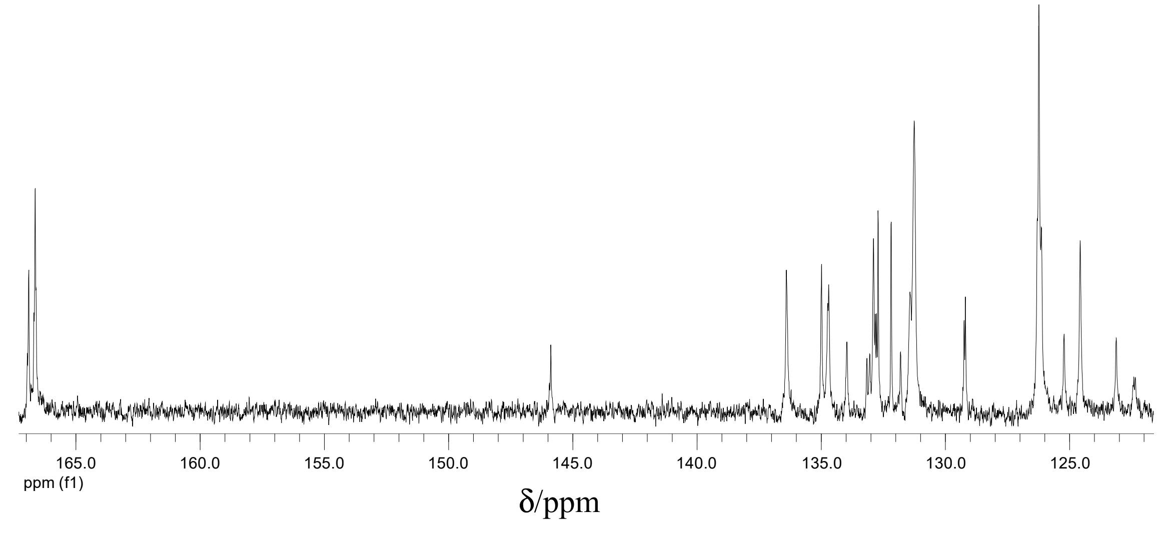

***Figure S17**. ¹³C-NMR for BPDA$_{3/1}$-6F copolymer.*

^{13}C- NMR, 75 MHz, $CDCl_3$:166.9 (C_f, C_r), 166.7 (C_g, C_o), 145.9 (C_p), 136.4 (C_b), 135.0 (C_e), 134.7 (C_c), 134.0 (C_s), 132.9 (C_u), 132.8 (C_q), 132.7 (C_l), 132.2 (C_g), 131.4 (C_i), 131.3 (C_k), 129.2 (C_a), 126.3 (C_j), 125.2 (C_t), 124.6 (C_d), 122.8 (C_v).

S2.4.^{1}H, ^{13}C-NMR for BPDA$_{1/1}$-6F copolymer

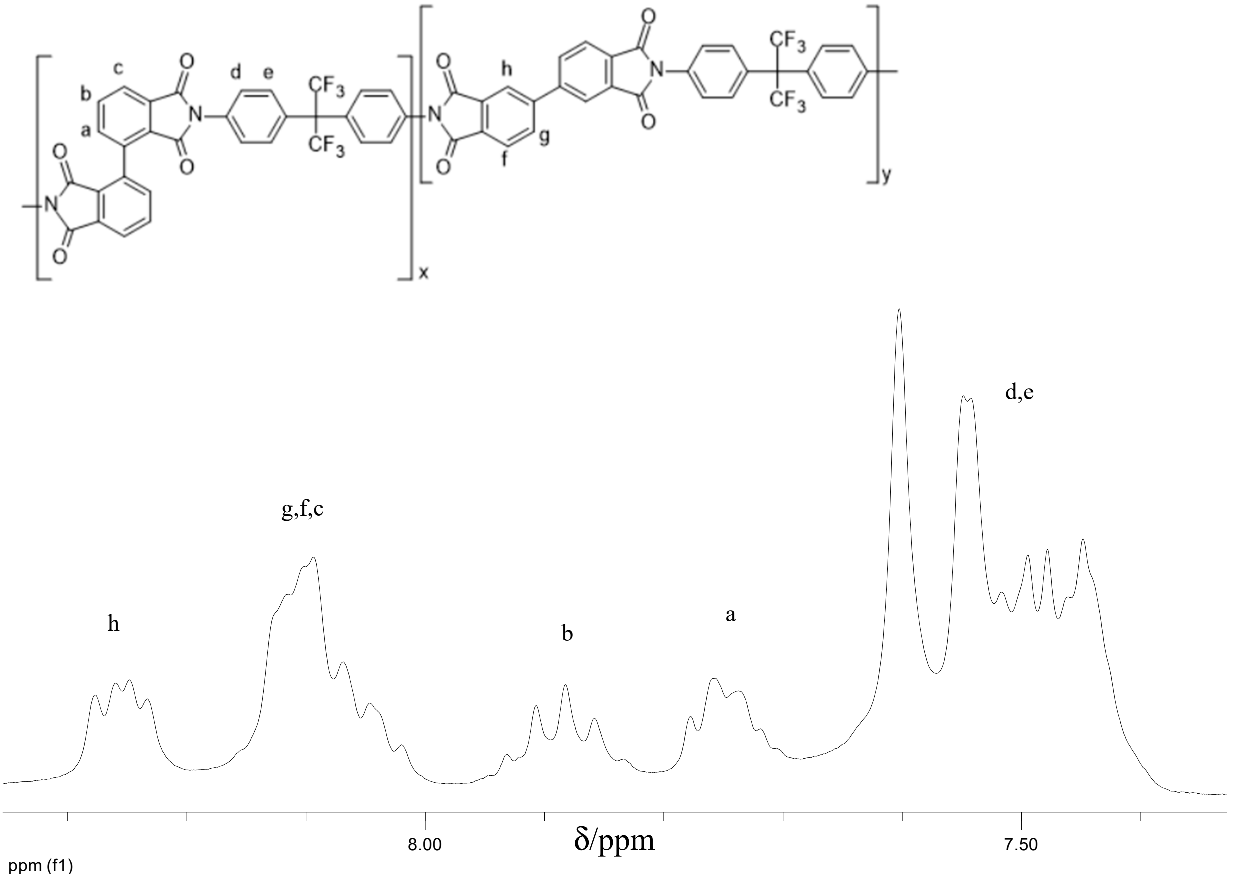

Figure S18. *^{1}H-NMR for BPDA$_{1/1}$-6F copolymer*

^{1}H- NMR, 300 MHz, $CDCl_3$: 8.26-8.10 (m, 2H, H_v), 8.09-7.97 (m, 6H, Ht-Hs-H_b), 7.96-7.83 (m, 2H, H_c), 7.81-7.68 (m, 2H, H_b), 7.59-7.35 (m, 16H, H_j, H_k).

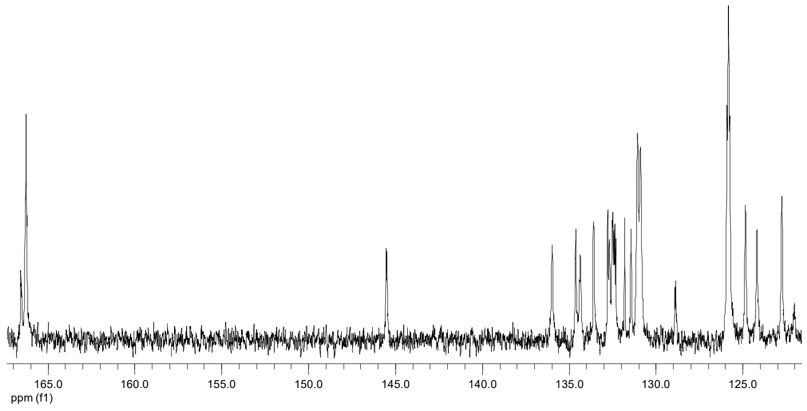

δ/ppm

Figure S19. *^{13}C-NMR for $BPDA_{1/1}$-6F copolymer.*

^{13}C- NMR, 75 MHz, $CDCl_3$:166.6 (C_f, C_r), 166.2 (C_g, C_o), 145.5 (C_p), 136.0 (C_b), 134.6 (C_e), 134.3 (C_c), 133.6 (C_s), 132.8 (C_u), 132.7 (C_q), 132.4 (C_l), 131.8 (C_g), 131.4 (C_i), 131.0 (C_k), 129.2 (C_a), 125.9 (C_j), 124.8 (C_t), 124.2 (C_d), 122.7 (C_v).

S2.5.^{1}H, ^{13}C-NMR for $BPDA_{1/3}$-6F copolymer

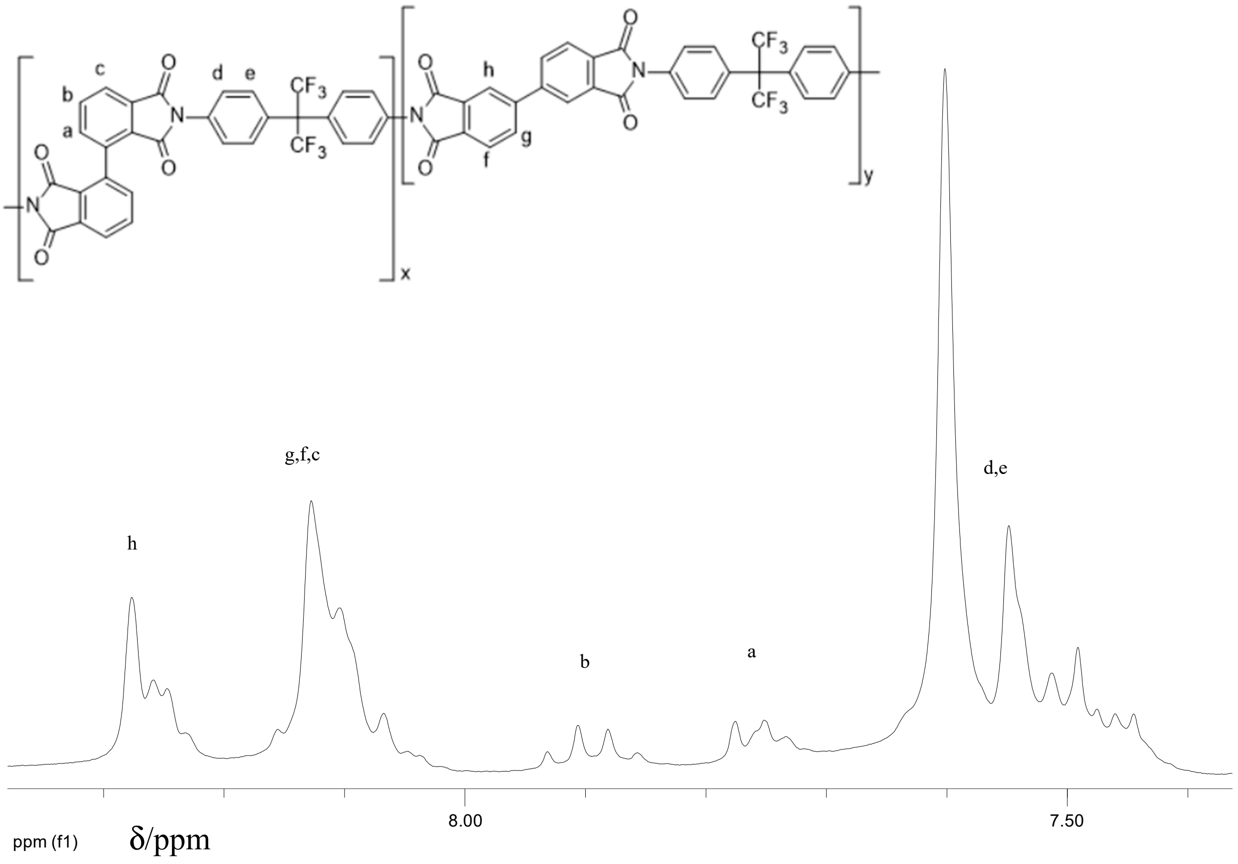

Figure S20*. ^{1}H-NMR for $BPDA_{1/3}$-6F copolymer*

^1H- NMR, 300 MHz, $CDCl_3$: 8.36-8.20 (m, 6H, H_v), 8.19-8.01 (m, 14H, H_t-H_s-H_b), 7.95-7.83 (m, $2H_c$, H_c), 7.82-7.62 (m, 2H, H_b), 7.60-7.37 (m, 32H, H_j, H_k).

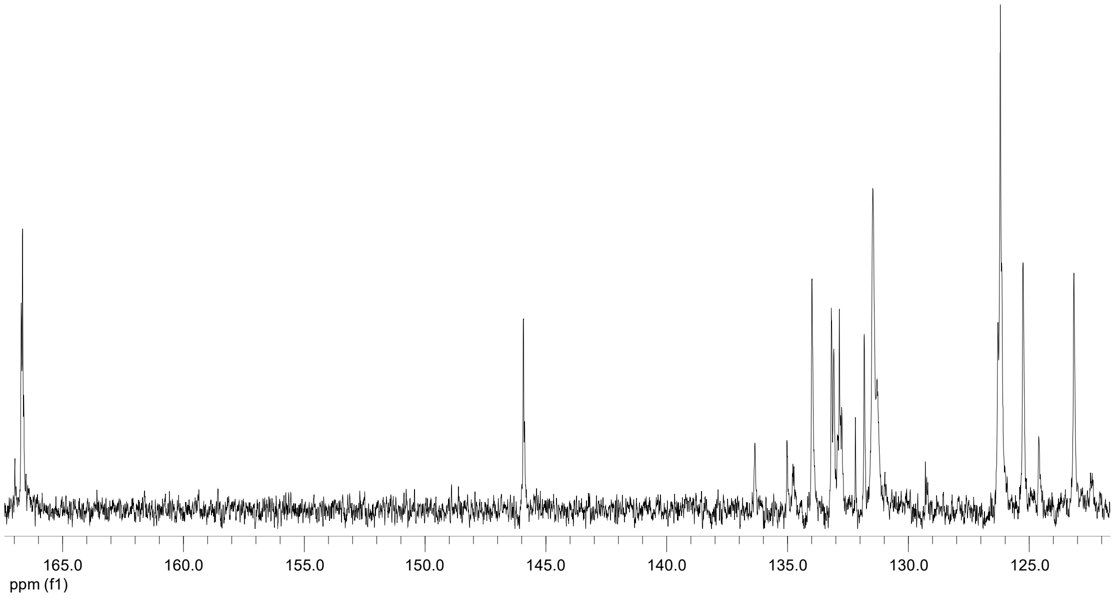

Figure S21. *^{13}C-NMR for BPDA$_{1/3}$-6F copolymer.*

^{13}C- NMR, $CDCl_3$:167.0 (C_f y C_r), 166.7 (C_g y C_o), 146.0 (C_p), 136.4 (C_b), 135.0 (C_e), 134.8 (C_c), 134.0 (C_s), 132.2 (C_u), 132.1 (C_q), 132.9 (C_l), 132.2 (C_g), 131.9 (C_i), 131.5 (C_k), 129.3 (C_a), 126.3 (C_j), 125.3 (C_t), 124.6 (C_d), 123.0 (C_v).

S3. Elemental microanalysis data

Table S1. *Elemental microanalysis data.*

**Polymer**	**%C**	**%H**	**%N**	**% Difference from the theoretical value**		
				**%C**	**%H**	**%N**
	62.74*	2.55*	4.72	*--------	--------	-------
***i*BPDA-6F**	56.14	2.22	4.62	10.52	12.94	2.12
**$BPDA_{3/1}$-6F**	53.63	2.04	4.28	14.52	20.00	9.32
**$BPDA_{1/1}$-6F**	59.25	2.03	3.68	5.56	20.39	22.03
**$BPDA_{1/3}$-6F**	59.76	2.27	4.67	4.75	10.98	1.06
**BPDA-6F**	57.14	2.06	4.30	8.93	19.22	8.90

*The shaded cells represent the theoretical % value.

S4. FTIR characterization of the polymers

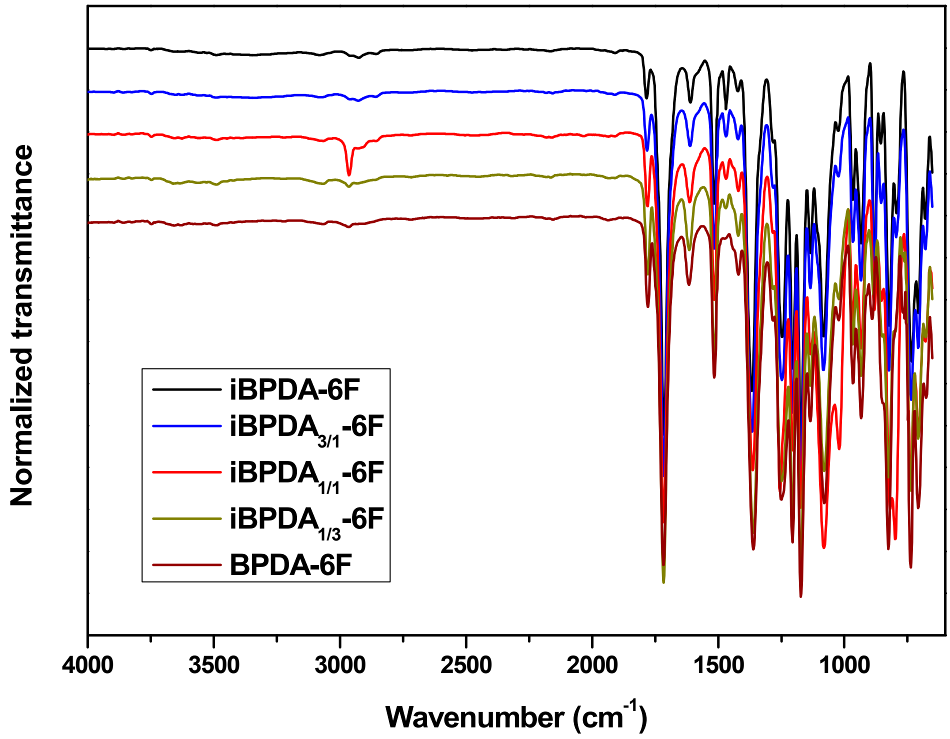

Figure S22. *FTIR spectra of polymers*

S5. Viscosities and molecular weights

Table S2. *Inherent viscosity values, molecular weights (M_n and M_w), and polydispersity values of polymers.*

**Polymer**	η_{inh} /dlg^{-1}	M_w	M_n	IP
**iBPDA-6F**	1.01	216000	128000	1.69
**iBPDA$_{3/1}$-6F**	1.56	290000	216000	1.34
**iBPDA$_{1/1}$-6F**	1.29	272000	193000	1.41
**iBPDA$_{1/3}$-6F**	1.98	208000	151000	1.38
**BPDA-6F**	2.41	263000	188000	1.40

S6. Solubility of isomeric polyimides

Table S3. *Solubility of polyimides*

	Solvent							
**Polymer**	**DMAc**	**Acetone**	**Ethanol**	**$CHCl_3$**	**DMF**	**THF**	**NMP**	***m*-cresol**
**iBPDA-6F**	++	-	-	++	++	++	++	++
**$iBPDA_{3/1}$-6F**	++	-	-	++	++	++	++	++
**$iBPDA_{1/1}$-6F**	++	-	-	++	++	++	++	++
**$iBPDA_{1/3}$-6F**	-	-	-	++	+	+	+	+
**BPDA-6F**	+	-	-	++	+	+	++	++

++: soluble, +: soluble in hot, -: not soluble

S7. Densities and fractional free volume of polymer films

Table S4. *Densities and Fractional Free Volume (FFV)*

Polymer	density (g/cm³)	V_0 (cm³/g)	V_0 (cm³/mol)	V_w (Å³/su)	V_w (cm³/mol)	FFV
**iBPDA-6F**	1.3968	0.7159	423.88	440.99	265.61	0.186
**iBPDA$_{3/1}$-6F**	1.3945	0.7171	424.59	440.90	265.55	0.187
**iBPDA$_{1/1}$-6F**	1.4015	0.7135	422.46	440.80	265.49	0.183
**iBPDA$_{1/3}$-6F**	1.3883	0.7203	426.49	440.71	265.44	0.190
**BPDA-6F**	1.3861	0.7215	427.20	440.61	265.38	0.192

su; polymer structural unit

S8. Thermal properties of polyimides, DSC and TGA

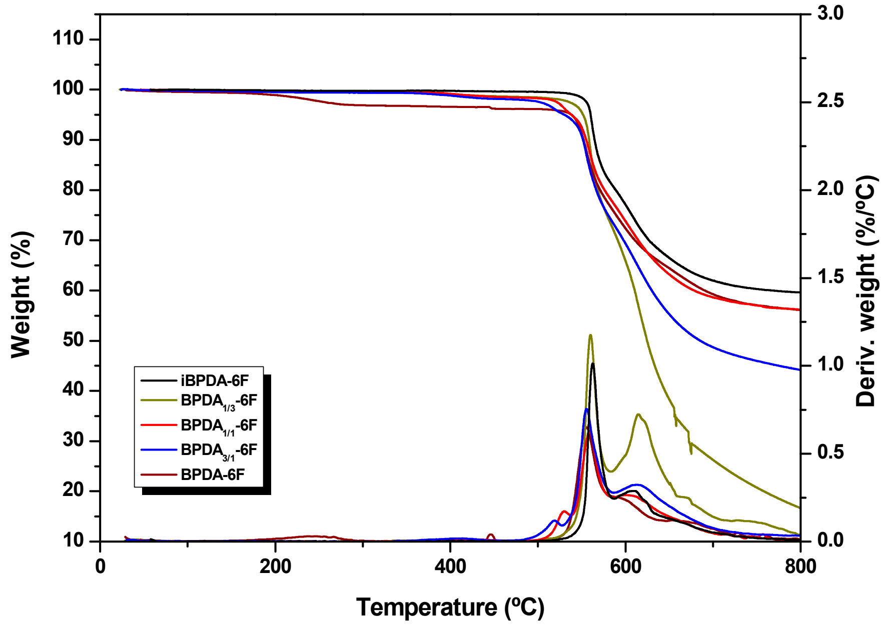

Figure S23. *TGA of polyimides*

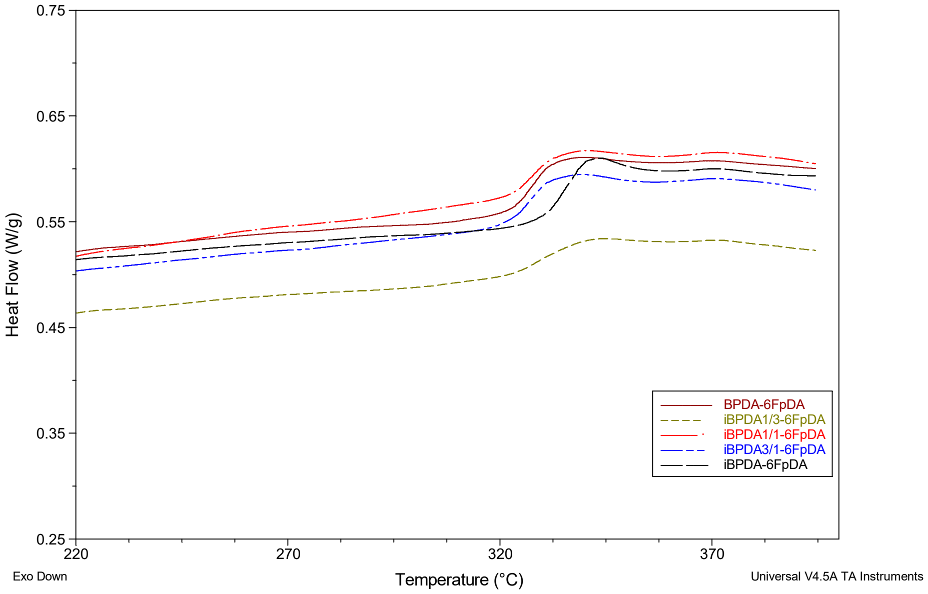

Figure S24. *DSC of polyimides*

S9. Mechanical properties of polyimides

Table S5. *Mechanical properties*

Polymer	Modulus (GPa)	Tensile strength (MPa)	Elongation at break (%)
**iBPDA-6F**	1.9 ± 0.1	49.6 ± 5.1	3.5 ± 0.5
**iBPDA$_{3/1}$-6F**	1.87 ± 0.07	79.3 ± 2.9	13.7 ± 3.6
**iBPDA$_{1/1}$-6F**	1.83 ± 0.15	95.5 ± 2.8	14.7 ± 1.4
**iBPDA$_{1/3}$-6F**	2.17 ± 0.06	90.0 ± 5.8	58.3 ± 3.4
**BPDA-6F**	2.07 ± 0.03	96.7 ± 13.0	29.2 ± 14.7

S10. Dynamomechanical properties

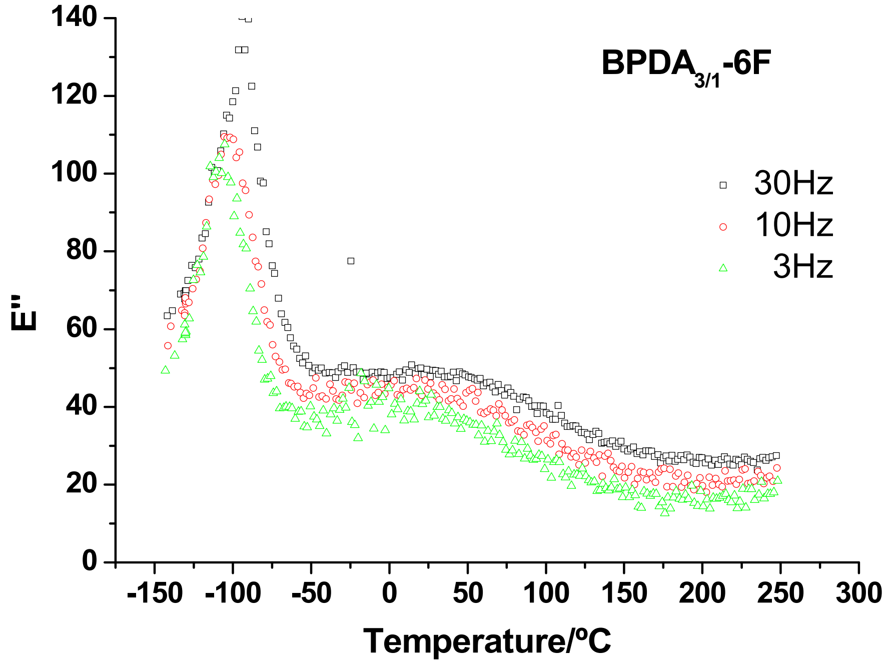

Figure S25. *E''vs T for BPDA$_{3/1}$-6F*

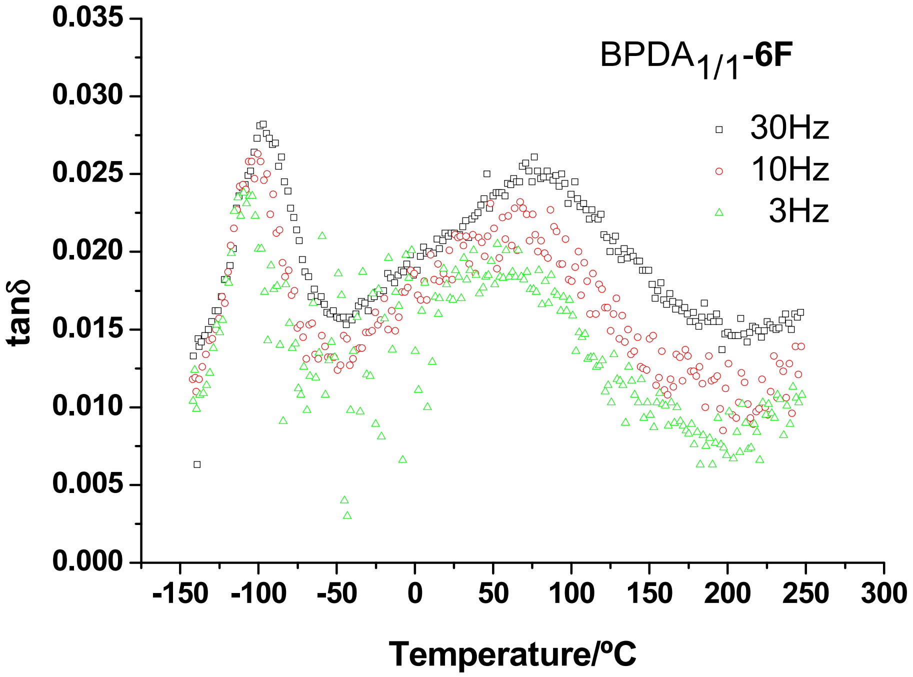

Figure S26. *E''vs T for BPDA$_{1/1}$-6F*

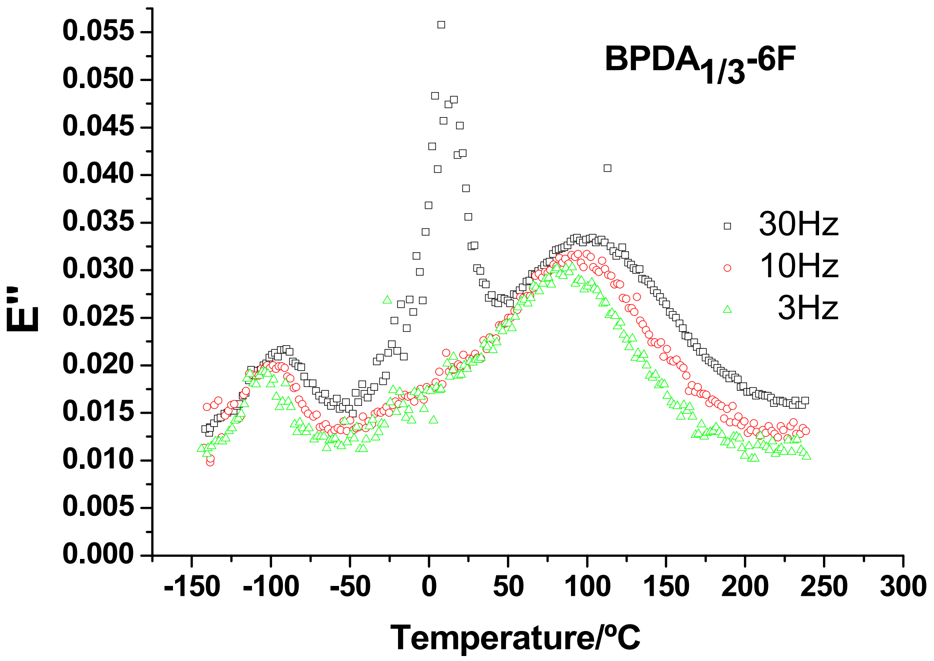

Figure S27. *E'' vs T for BPDA$_{1/3}$-6F*

S11. Activation energy and temperature data

Table S6. *Activation energy and peak temperature of the maximum of the DMTA graph at 10Hz.*

Polyimide	E_a/Kj mol^{-1}		T/ºC (υ =10Hz)	
	Peak I	Peak II	Peak I	Peak II
iBPDA-6F	155	--------	-103.4	-----
iBPDA$_{3/1}$-6F	78	94	-102.6	17.2
iBPDA$_{1/1}$-6F	106	148	-104.5	48.3
iBPDA$_{1/3}$-6F	108	--------	-102.6	82.6
BPDA-6F	66	231	-99.7	101.7

S12. Permeabilities, Diffusivities and gas Selectivity of membranes

Table S7. *Diffusion and Solubility Coefficients, D (cm^2/s)·10^8 and S (cm^3/cm^3·cmHg)·10^3*

Polymer	DO_2	DN_2	DCH_4	DCO_2	SO_2	SN_2	SCH_4	SCO_2
**iBPDA-6F**	10.60	1.99	0.89	4.21	12.66	13.83	16.27	140.20
**iBPDA$_{3/1}$-6F**	5.03	1.46	0.51	2.58	17.31	12.86	31.14	154.12
**iBPDA$_{1/1}$-6F**	4.32	0.94	0.37	2.54	17.79	18.63	30.74	149.57
**iBPDA$_{1/3}$-6F**	5.01	1.09	0.26	2.09	14.00	14.45	44.21	144.69
**BPDA-6F**	4.66	1.02	0.28	1.95	12.70	12.10	27.14	136.45

Table S8. *Ideal selectivities (α) and ideal diffusivities (α_D)*

Polymer	$\alpha O_2/N_2$	$\alpha CO_2/O_2$	$\alpha CO_2/N_2$	$\alpha CO_2/CH_4$	$\delta O_2/N_2$	$\delta CO_2/O_2$	$\delta CO_2/N_2$	$\delta CO_2/CH_4$
**iBPDA-6F**	4.9	4.4	21.4	40.6	5.3	0.4	2.1	4.7
**iBPDA$_{3/1}$-6F**	4.7	4.6	21.2	24.8	3.5	0.5	1.8	5.0
**iBPDA$_{1/1}$-6F**	4.4	4.9	21.8	33.0	4.6	0.6	2.7	6.8
**iBPDA$_{1/3}$-6F**	4.4	4.3	19.2	26.3	4.6	0.4	1.9	8.0
**BPDA-6F**	4.8	4.5	21.4	34.9	4.6	0.4	1.9	6.9

S13. Calculation of errors for gas permeability, gas diffusivity, and gas solubility

In order to calculate the error that affects each of the evaluated parameters, permeability and diffusivity, the errors made in the measurement must be previously known. In the calculation of permeability P, the measurement errors of the membrane thickness (e_l), the pressure of the high zone (e_p) and the slope of the permeation graphs ($e_{(dp/dt)}$) affect the calculation of permeability.

$$dP = \left|(\partial P/\partial l)dl\right| + \left|(\partial P/\partial p_o)dp_o\right| + \left|(\partial P/\partial(dp/dt))d(dp/dt)\right| =$$
$$= \left|(\partial P/\partial l)e_l\right| + \left|(\partial P/\partial p_o)e_{p_o}\right| + \left|(\partial P/\partial(dp/dt))e_{(dp/dt)}\right|$$

where

$$P = 0.128\frac{l}{P_0}\frac{dp}{dt}\cdot 10^{10}$$
$$e_l = \pm 2\mu m$$
$$e_{p_o} = \pm 0.001 bar$$
$$e_{(dp/dt)} = \pm e_B$$

where e_B is the error given by the linear least squares fit in each case. In all cases, the relative error did not exceed 8% for the permeability calculations.

As for the value of the diffusion coefficient D, we proceed in the same way:

$$dD = \left|(\partial D/\partial l)dl\right| + \left|(\partial D/\partial A)dA\right| + \left|(\partial D/\partial B)dB\right| =$$
$$= \left|(\partial P/\partial l)e_l\right| + \left|(\partial P/\partial A)e_A\right| + \left|(\partial P/\partial B)e_B\right|$$

where

$$D = \frac{l^2}{6\Theta} = \frac{l^2}{6(-A/B)}$$
$$e_l = \pm 0.0002 cm$$

where e_A *y* e_B are the errors given by the linear least square's adjustment in each case, where A is the value of the ordinate at the origin of the regression. In no case did the relative error exceed 7%.

According to the equation that defines solubility as a function of permeability and solubility coefficient (S=P/D), the error associated with this parameter can be considered as the sum of the relative errors of P and D, and will therefore have a value of less than 15%.
